# Using modeling and scenario analysis to support evidence-based health workforce strategic planning in Malawi

**DOI:** 10.1186/s12960-022-00730-3

**Published:** 2022-04-18

**Authors:** Leslie Berman, Margaret L. Prust, Agnes Maungena Mononga, Patrick Boko, Macfarlane Magombo, Mihereteab Teshome, Levison Nkhoma, Grace Namaganda, Duff Msukwa, Andrews Gunda

**Affiliations:** 1 Clinton Health Access Initiative, Inc. (CHAI) Malawi, Lilongwe, Malawi; 2 Analytics and Implementation Research Team, Clinton Health Access Initiative, Inc. (CHAI), Boston, MA USA; 3 Department of Human Resources Management and Development, Ministry of Health, Lilongwe, Malawi; 4 Human Resources for Health 2030 (HRH2030), Chemonics International, Lilongwe, Malawi

**Keywords:** Human resources for health, Malawi, Strategic planning, Workforce development, Recruitment

## Abstract

**Background:**

A well-trained and equitably distributed workforce is critical to a functioning health system. As workforce interventions are costly and time-intensive, investing appropriately in strengthening the health workforce requires an evidence-based approach to target efforts to increase the number of health workers, deploy health workers where they are most needed, and optimize the use of existing health workers. This paper describes the Malawi Ministry of Health (MoH) and collaborators’ data-driven approach to designing strategies in the Human Resources for Health Strategic Plan (HRH SP) 2018–2022.

**Methods:**

Three modelling exercises were completed using available data in Malawi. Staff data from districts, central hospitals, and headquarters, and enrollment data from all health training institutions were collected between October 2017 and February 2018. A vacancy analysis was conducted to compare current staffing levels against established posts (the targeted number of positions to be filled, by cadre and work location). A training pipeline model was developed to project the future available workforce, and a demand-based Workforce Optimization Model was used to estimate optimal staffing to meet current levels of service utilization.

**Results:**

As of 2017, 55% of established posts were filled, with an average of 1.49 health professional staff per 1000 population, and with substantial variation in the number of staff per population by district. With current levels of health worker training, Malawi is projected to meet its establishment targets in 2030 but will not meet the WHO standard of 4.45 health workers per 1000 population by 2040. A combined intervention reducing attrition, increasing absorption, and doubling training enrollments would allow the establishment to be met by 2023 and the WHO target to be met by 2036. The Workforce Optimization Model shows a gap of 7374 health workers to optimally deliver services at current utilization rates, with the largest gaps among nursing and midwifery officers and pharmacists.

**Conclusions:**

Given the time and significant financial investment required to train and deploy health workers, evidence needs to be carefully considered in designing a national HRH SP. The results of these analyses directly informed Malawi’s HRH SP 2018–2022 and have subsequently been used in numerous planning processes and investment cases in Malawi. This paper provides a practical methodology for evidence-based HRH strategic planning and highlights the importance of strengthening HRH data systems for improved workforce decision-making.

**Supplementary Information:**

The online version contains supplementary material available at 10.1186/s12960-022-00730-3.

## Background

The health workforce is an essential building block for a functioning health system and achievement of universal health coverage [1]. The Government of Malawi has made significant investments in its health workforce since 2006 with the introduction of the Emergency Human Resources Programme (EHRP), which led to the scale up of health workers in 11 priority cadres by 53% from 5453 at baseline, to 8369 by 2009 [2]. Government commitment has been complemented by significant donor investment in the health workforce. Government and donors invested USD 257 357 000 in health worker salaries and USD 58 487 000 in pre-service education of health workers between 2012 and 2016 [3].

Despite these investments, review of achievements during Malawi’s first Human Resources for Health Strategic Plan (HRH SP) 2012–2016 highlighted widespread and persistent workforce challenges [4]. At the close of the first HRH strategic planning period, the country continued to face significant health worker shortages, over-concentration of health workers in urban areas, insufficient and misaligned training of new health workers to fill large staffing gaps, and limited capacity to hire newly trained health workers due to budgetary constraints [4–6].

Health workforce interventions can be costly, take many years to show results, and require cooperation of numerous stakeholders [1, 7]. Investing appropriately in strengthening the health workforce requires an evidence-based approach to HRH planning to ensure limited resources are efficiently utilized towards targeted efforts to increase the number of health workers, deploy health workers where they are most needed, and optimize the use of existing health workers.

Demand-based approaches to estimating workforce requirements offer low- and middle-income countries (LMICs) a practical methodology for medium-term target setting. Demand-based target setting considers variation in the use of services across geographies and complexities of service delivery by different cadres of health workers, which are missed by target setting methods using population ratios or facility-based standards [8]. While needs-based estimation can offer idealized targets, results often suggest health worker volumes that are unrealistic for setting national targets in LMICs [8–10].

The World Health Organization introduced the Workload Indicators of Staffing Need (WISN) model in 1998, which uses health worker workload data to determine workforce requirements to deliver a specific package of services [8]. The model has since been widely used to determine volume and cadre of staff required in selected health facilities [11–13]. In 2009, the Clinton Health Access Initiative (CHAI) and the Zambian Ministry of Health (MoH) first developed the Workforce Optimization Model (WFOM), which has since been used in Malawi, Lesotho, Liberia, and Eswatini [14]. The model uses health worker time-motion observations alongside service delivery data to generate national workforce targets by cadre, facility type, and region. The WFOM and WISN are functionally similar, although the WFOM can be conducted in Stata using a simplified approach to inputting data, and has functionality to allow comparison of various policy scenarios.

The pre-service education system in LMICs is largely responsible for the overall supply of health workers. Pre-service education planning is critical to determining overall vacancy rates, distribution of health workers, and types of care available in-country [15]. Nonetheless, pre-service education planning is frequently overlooked by health system planners [15, 16]. Efforts to better align health worker supply with health system demand to ensure future availability of the health workforce requires an analysis of the pre-service training pipeline. This paper describes a pre-service training model developed by CHAI to forecast training production and projected workforce volumes for prioritized cadres under current and simulated scenarios.

While HRH modelling is a critical tool for evidence-based HRH decision-making [1, 17], these approaches are rarely used explicitly for the purposes of informing national HRH strategic planning in LMICs [18, 19]. A recent review of health workforce analytics in 20 LMICs did not identify any examples of countries that had institutionalized routine use of evidence-based methods for workforce planning [20]. Against this background, the Malawi MoH and collaborators decided to undertake a practical and evidence-based approach to developing its successor HRH Strategic Plan (HRH SP) 2018–2022. This paper describes the process of developing a data-driven HRH SP, including identifying information gaps, designing a targeted data collection and analysis plan, developing practical and flexible HRH models to conduct workforce scenario analyses, and ultimately utilizing model results to develop interventions in Malawi’s HRH SP 2018–2022.

## Methods

This section explains the approach to data collection and to executing the three key analyses presented in Malawi’s HRH SP 2018–2022: a vacancy analysis, pipeline model, and Workforce Optimization Model.

### Data collection

The Government of Malawi is the main provider of health services, which are delivered through a network of community health, primary, secondary, and tertiary facilities. Under decentralization, district governments administer community, primary, and secondary services across the country. Four centrally managed hospitals provide tertiary services in Malawi’s four main cities in each of the country’s regions (southwest, southeast, central, and north). A basic package of essential health services is offered in the public sector free-of-charge at the point of service delivery. In addition to MoH, the Christian Health Association of Malawi (CHAM), a large, faith-based non-governmental health care provider, provides approximately 29% of Malawi’s healthcare through service level agreements [4].

Primary workforce data was collected between October 2017 and February 2018. MoH headquarters, central hospitals, and districts provided “staff return” data, an Excel sheet that HR officers update monthly that lists personal details of all health workers in their respective catchment areas. CHAM provided similar staff returns for all CHAM facilities. Data on number of community health workers was provided by the MoH Community Health Services Unit from their December 2017 headcount of all Disease Control and Surveillance Assistants (DCSAs) and Senior DCSAs. Pre-service education data was collected from all 20 public, CHAM, and private pre-service education institutions in Malawi using a standard data collection tool that asked questions about enrollment, graduation, staffing, and infrastructure. Program data collection was accompanied by stakeholder consultations at national and sub-national level led by MoH, with training councils, regulatory bodies, health workers, donors, and HRH implementers. All primary data was deidentified, consolidated from standard government program data, and analyzed by government for strategic planning purposes. Therefore, Institutional Review Board approval was not required.

### Vacancy methodology and analysis

The purpose of the vacancy analysis was to compare current staffing levels against officially established posts (the targeted number of positions to be filled, by cadre and work location). MoH and CHAM use a facility-based staffing standard, which assigns targets for specific numbers of health worker by cadre according to health facility type.

The total number of staff by cadre and district was calculated using the staff return data. The vacancy analysis was conducted in Excel by comparing staff return data against the 2014/2015 MoH and 2017 CHAM list of established posts. Comparisons were conducted at the national, central hospital, and district levels, as well as for specific cadres. There was wide variation in position titles, and therefore, data were cleaned and standardized with input from the MoH Department of Human Resources Management and Development. The analysis focused on 22 unique sub-cadres that fall within nine broader cadre groupings. In addition, health worker to population ratios were calculated using the staff return data compared to the Malawi National Statistics Office population projections [21].

### Pipeline methodology and analysis

A training pipeline model was developed in Excel to estimate the number of health workers expected to be available in the future workforce based on current training, recruitment, and retention trends, and under various intervention scenarios. The model takes as its foundation the current workforce and adds to this the expected future inflow of health workers from training institutions and those hired from abroad, and deducts the projected future outflow due to retirement, attrition, or health workers who have gone back to school for training. The model uses the following equation to calculate future available workforce on an annual basis:

$$\left[1\right]\mathrm{ Current\,workforce}+\left[2\right]\mathrm{ Future\,Inflow}-\left[3\right]\mathrm{ Future\,Outflow}=\left[4\right]\mathrm{ Future\,Available\,Workforce}$$

where, 1 = Current staff as reflected in MoH and CHAM staff returns.

2 = (Enrolled students) − (Non-graduating students) − (Graduates not hired) + (Health workers hired from abroad).

3 = (Retired) + (Involuntary attrition) + (Voluntary attrition) + (Study leave).

The model was run using baseline data and workforce assumptions presented in Table 1 below to calculate the projected future available workforce in the non-intervention scenario, as well as in four intervention scenarios to reduce attrition, increase absorption, increase training enrollment, and a combined scenario incorporating the three prior interventions. Further details on model data sources and assumptions are provided in Additional file 1.

**Table 1:** Baseline and intervention scenarios in the pipeline model

	Baseline	Intervention 1: Reduced attrition	Intervention 2: Increased health worker absorption	Intervention 3: Increased training enrollment	Intervention 4: Combination of interventions 1–3
Current Workforce	Total number of health workers in 2017 as per MoH and CHAM staff return data and MoH headcount of DCSAs between Jun. and Dec. 2017	Baseline Scenario	Baseline Scenario	Baseline Scenario	Baseline Scenario
Future Inflow	Enrollment rates collected at all training colleges remain constant, except DCSAs, where enrollment increases to 7000 DCSAs by 2022^1^ Graduation rates calculated for each cadre based on data from training colleges ranges from 75 to 100% and remains constant Eligibility for hiring uses 2017 licensing exam pass rates and remains constant^2^; 100% eligibility for all cadres without exams 50% absorption rate calculated from MoH and CHAM data on new hires compared with number of eligible graduates; 100% absorption of DCSAs^3^ Hired abroad calculated from staff return, and remains constant	Baseline Scenario	Absorption of graduates increases from 50 to 75% by 2018, except DCSAs DCSAs absorbed 100% annually^3^	Doubling training enrollment by 2020 and again by 2030, except DCSAs DCSA enrollment remains constant^4^	Absorption of graduates increases from 50 to 75% by 2018, except DCSAs Doubling training enrollment by 2020 and again by 2030, except DCSAs DCSAs absorbed 100% annually and enrollment remains constant^3, 4^
Future Outflow	7% attrition, including﻿: 1% retirement rate calculated from staff returns, 2% involuntary and 3% voluntary attrition, and 1% study leave^5^	Voluntary attrition reduced incrementally from 3 to 1% by 2022	Baseline Scenario	Baseline Scenario	Voluntary attrition reduced incrementally from 3 to 1% by 2022

^1^Training increases according to the MoH National Community Health Strategy

^2^See Additional file 1 for specific pass rates by cadre

^3^DCSAs are specifically trained and hired for vacant posts

^4^Based on projections from the MoH Community Health Services Unit

^5^See Additional file 1 for assumptions on voluntary and involuntary attrition

The future available workforce in each scenario was then compared to two workforce targets: the MoH and CHAM establishment, and the WHO recommendation of 4.45 skilled health workers per 1000 population to meet the Sustainable Development Goals [22]. The establishment target was held constant overtime, whereas the WHO population-based target increased overtime using the Malawi National Statistics Office population projections [21].

### Workforce optimization model (WFOM) methodology and analysis

The WFOM was used to model optimal workforce requirements. The model uses information on health service coverage and health worker time required to perform those services to calculate demand-based workforce requirements using the following equation:

$$\frac{\left(\left[1\right]\mathrm{Number\,of\,services\,provided}*\left[2\right]\mathrm{Time\,required\,from\,each\,cadre\,per\,service}\right)}{\left[3\right]\mathrm{Time\,available\,for\,patient\,facing\,activities\,per\,health\,worker\,per\,year}}=\left[4\right]\mathrm{Health\,worker\,requirement}$$

where, 1 = Number of times each activity is performed at each facility per year.

2 = (Number of minutes spent on each activity per health worker for the given facility type) * (Proportion of time different cadres perform each activity in the given facility type).

3 = [(Number days per year) − (Public holidays) − (Vacation days) − (Sick days) − (Study leave days) − (Average maternity leave across cadre)] * [(Patient-facing hours per day) * (Number of minutes in an hour)].

Health services included in the model were based on the essential health package elaborated in Malawi’s Health Sector Strategic Plan II 2017–2022 [4]. Additional file 1 provides a complete list of services included in the model. Data on volume of services provided was taken from 2016 data routinely collected by the government including through the District Health Information Software (DHIS2) system, MoH HIV Quarterly Supervision Reports, the national HIV Laboratory Information Management System, and laboratory system data. Surgery data was directly collected from Kamuzu Central Hospital as it is not available in any central reporting system. Six hundred MoH and CHAM facilities with sufficient available data were included in the model.

The results show the optimal number of health workers needed to provide the current level of service utilization as of 2016 with optimal quality (i.e., the correct cadre providing the service and taking the recommended amount of time per patient). Given significant national workforce vacancies and limited financial resources to hire and train new health workers, this scenario was developed to provide the government with an attainable medium-term target to meet current levels of service utilization and optimize the existing health workforce.

Health worker time required to provide services was generated in minutes using previously collected time-motion estimates, where facility-based observations of provider–patient encounters were timed with a stopwatch [23, 24]. These data were presented and validated during an expert clinical consultation organized by MoH in November 2017. Health worker roles in the provision of specific services were allocated to each cadre at each level of the health system based on health worker job descriptions and validated during the expert consultation. The final activity times used in the model by cadre and facility type, as well as additional details on data collection for time-motion estimates, can be found in Additional file 1.

Health workers’ available patient-facing time was determined using Malawi’s Public Service Regulations, which elaborate staff entitlements [25]. Public service information was complemented by previously generated assumptions about the amount of time that health workers spend on administrative and other non-patient facing tasks [23]. These assumptions were validated during the November 2017 expert consultation. The details on patient facing time assumptions can be found in Additional file 1.

The results of the model were then compared to the establishment at the district level and filled positions as per the vacancy analysis to calculate gaps between filled, optimized demand-based targets, and established positions by district, cadre, and facility type. The model does not include DCSAs, since recruitment targets for this cadre are defined in the National Community Health Strategy, and the optimization model is primarily designed to consider service use in health facilities rather than community-based service delivery. As data were not available for services provided by certain cadres included in the vacancy analysis, such as mental health, dental, radiography, and nutrition, these cadres were not included in the WFOM.

## Results

In May 2018, the Government of Malawi successfully launched the HRH SP 2018–2022, which includes the analyses described here [26].

### Vacancy analysis results

**Fig. 1:**
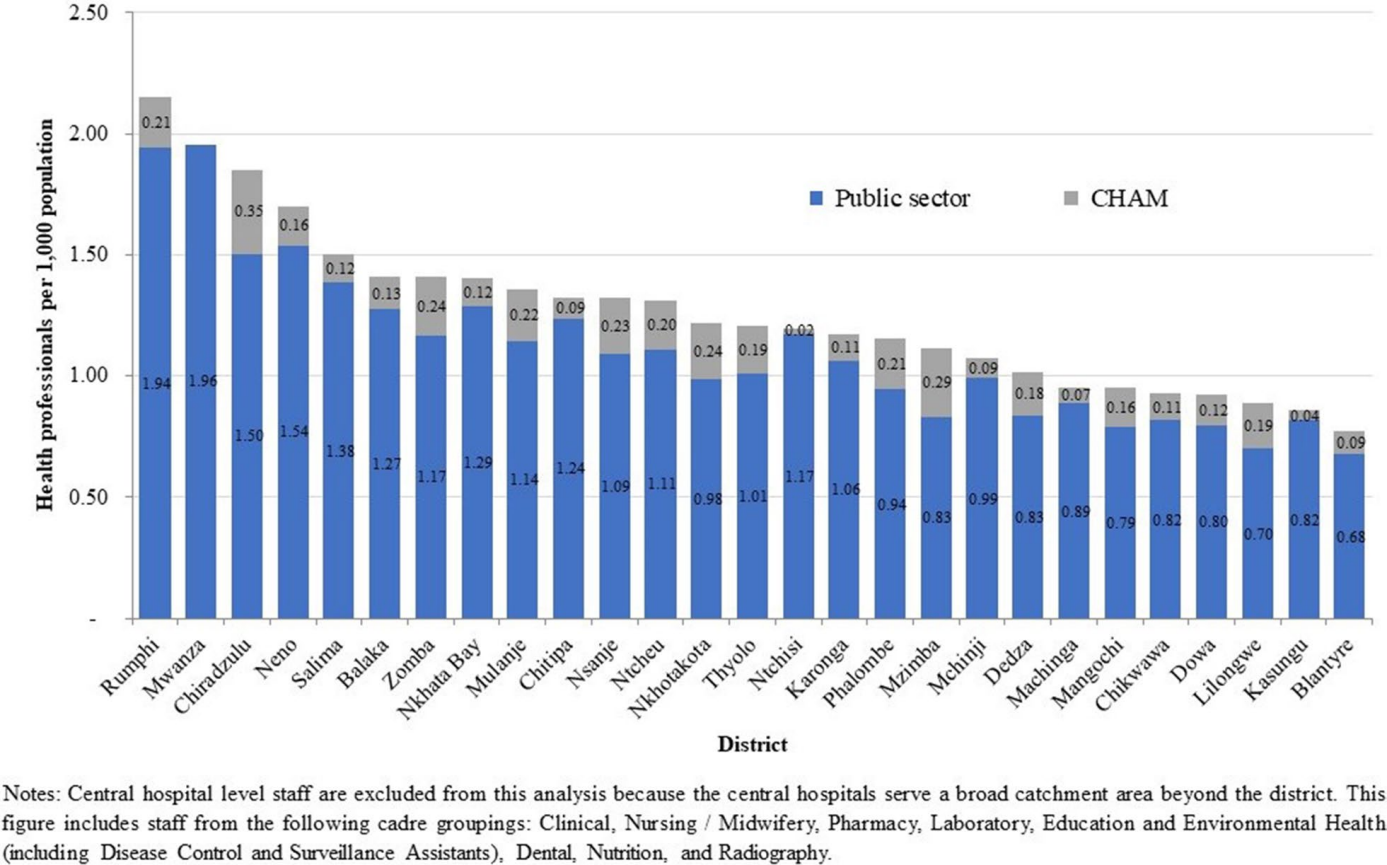
Frontline health workers employed by MoH and CHAM per 1000 population 2017

In 2017 the MoH and CHAM employed 37 926 staff in the health sector, of which 21 128 (56%) were frontline health workers. Frontline workers include all cadre groups listed in Table 2 below. There were 1.49 frontline health workers per 1000 population, with substantial variation in the number of staff per population by district (see Fig. 1).

**Table 2:** Number and percent of frontline health worker posts filled by sub-cadre, MoH and CHAM 2017

Cadre	Sub-cadre	Current staff	Establishment	Percentage of posts filled (%)
Clinical	Medical Officer/Specialist	564	784	72
	Clinical Officer/Technician	1311	3956	33
	Medical Assistant	1213	1739	70
Nursing/Midwifery	Nursing/Midwifery Officer	992	1498	66
	Nurse Midwife Technician	4467	12 701	35
	Community Midwifery Assistant	209	208	100
Pharmacy	Pharmacy Officer	66	98	67
	Pharmacy Technician	159	832	19
	Pharmacy Assistant	81	472	17
Laboratory	Laboratory Officer	94	83	113
	Laboratory Technician	351	821	43
	Laboratory Assistant	116	610	19
Education and Environmental Health	Education/Environmental Health Officer	591	1081	55
	Disease Control and Surveillance Assistant	10 085	10 099	100
Dental	Dental Officer	31	104	30
	Dental Therapist	123	681	18
Nutrition	Nutrition Officer	36	63	57
	Home Craft Worker	470	1922	24
Radiography	Radiographer	75	137	55
	Radiography Technician	94	204	46
	Total	21 128	38 093	55

There are a total of 62 269 positions in the MoH and CHAM establishment, of which 38 093 (61%) are frontline health worker positions. As of 2017, 55% of frontline health worker posts were filled. Vacancy rates varied greatly by cadre, with more senior sub-cadres with fewer established posts having lower vacancy rates than less specialized, lower level cadres with higher numbers of established posts (see Table 2).

Vacancy rates also varied by district (see Table 3). As highlighted in Fig. 1, Malawi’s two most populous districts with the country’s largest urban centers, Lilongwe and Blantyre, had among the lowest number of health workers per population excluding central hospital staff (0.89 and 0.77 health workers per 1000 population, respectively). However, these districts had among the lowest vacancy rates according to establishment targets (87% and 82% of posts filled, respectively). Central hospitals in Lilongwe and Blantyre, which serve the central and southwest region, had the overall lowest vacancy rates (93 and 100% of posts filled, respectively). These central hospitals also had substantially lower vacancy rates than the nation’s two other central hospitals, Mzuzu Central Hospital serving the northern region, and Zomba Central Hospital serving the southeastern region (48% and 44% of posts filled, respectively).

**Table 3:** Percentage of establishment posts filled by district and central hospital, MoH and CHAM 2017

Region	District	Current Staff	Establishment	Percentage of posts filled (%)
Central (62% of posts filled)	Dedza	782	1234	63
	Dowa	766	1161	66
	Kasungu	767	1317	58
	Lilongwe	2227	2560	87
	Mchinji	681	1103	62
	Nkhotakota	493	1095	45
	Ntcheu	793	1239	64
	Ntchisi	366	859	43
	Salima	669	943	71
North (44% of posts filled)	Chitipa	303	830	37
	Karonga	420	1028	41
	Mzimba	1346	2054	66
	Nkhata Bay	418	1213	34
	Rumphi	474	1116	42
South (58% of posts filled)	Balaka	596	911	65
	Blantyre	1058	1293	82
	Chikwawa	525	1081	49
	Chiradzulu	606	987	61
	Machinga	617	962	64
	Mangochi	1038	1492	70
	Mulanje	799	1403	57
	Mwanza	210	640	33
	Neno	282	795	35
	Nsanje	391	1068	37
	Phalombe	455	958	47
	Thyolo	803	1159	69
	Zomba	1186	1424	83
Central	Headquarters	174	3536	5
Central	Kamuzu Central Hospital	610	653	93
North	Mzuzu Central Hospital	313	650	48
Southwest	Queen Elizabeth Central Hospital	676	679	100
Southeast	Zomba Central Hospital	284	650	44
	Total	21 128	38 093	55

Malawi’s less populous northern region had the highest regional vacancy rate (44% of posts filled), compared with the southern region (58%) and central region (62%), excluding headquarters and central hospitals. All districts in the northern region had more than 50% of their posts vacant, except for Mzimba district, the northern region’s most populous district with the region’s only urban area.

### Pipeline model results

**Fig. 2:**
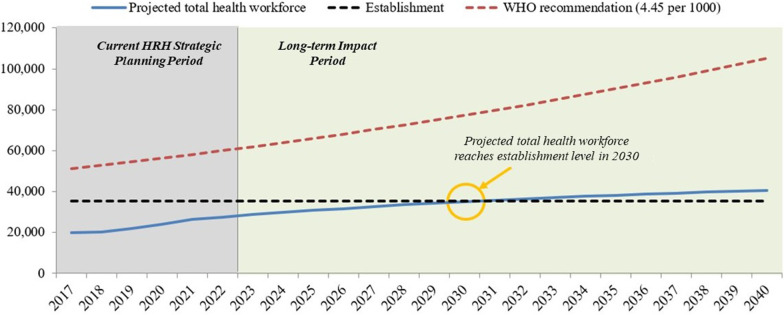
Projected health workforce compared to establishment and WHO recommended health workforce targets 2017–2040

Figure 2 shows the results of the pipeline model in the non-intervention scenario. It illustrates the total, annual projected available health workforce for all cadres through 2040 if baseline trends continue. Holding all parameters constant, Malawi would have sufficient heath workers available to meet its establishment levels in 2030, but is not projected to meet the WHO standard by 2040. At the end of the strategic planning period in 2022, there is expected to be a gap of 8978 health workers between the projected available health workforce and the establishment, with 75% of the establishment filled, and a gap of 33 643 health workers between the projected workforce and WHO targets.

**Fig. 3:**
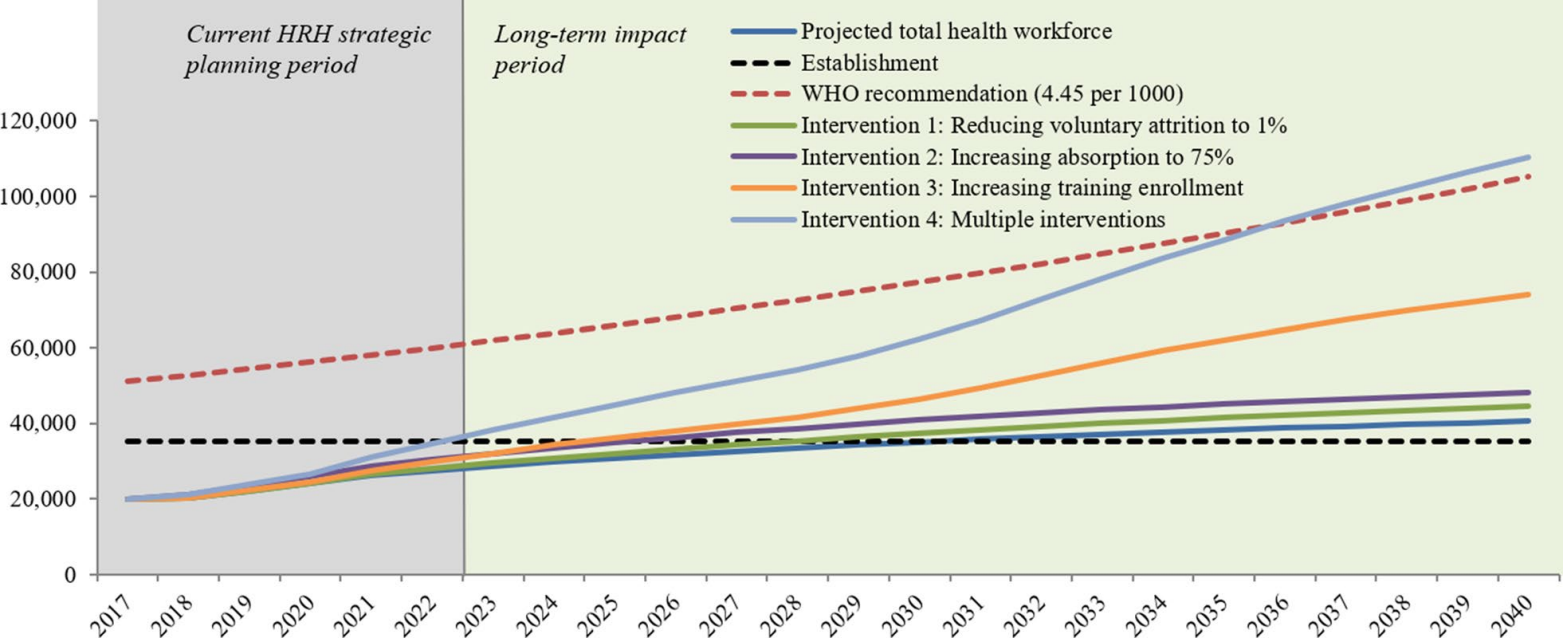
Impact of intervention scenarios on size of workforce for all cadres through 2040

Figure 3 shows the four intervention scenarios modelled to increase the production, recruitment, and retention of health workers to minimize the gap between the projected and required workforce. In Intervention 1, which reduces voluntary attrition, the projected workforce would meet the establishment target by 2028. In Intervention 2, which increases absorption of graduates, the projected workforce would meet the establishment by 2026. In Intervention 3, which increases pre-service training enrollment, the projected workforce would meet the establishment by 2025. In Intervention 4, the composite of all three preceding interventions, the projected workforce would meet the establishment in 2023, 7 years earlier than the non-intervention scenario. Intervention 4 is the only scenario where the WHO target would be achieved before 2040.

### Workforce optimization model results

**Fig. 4:**
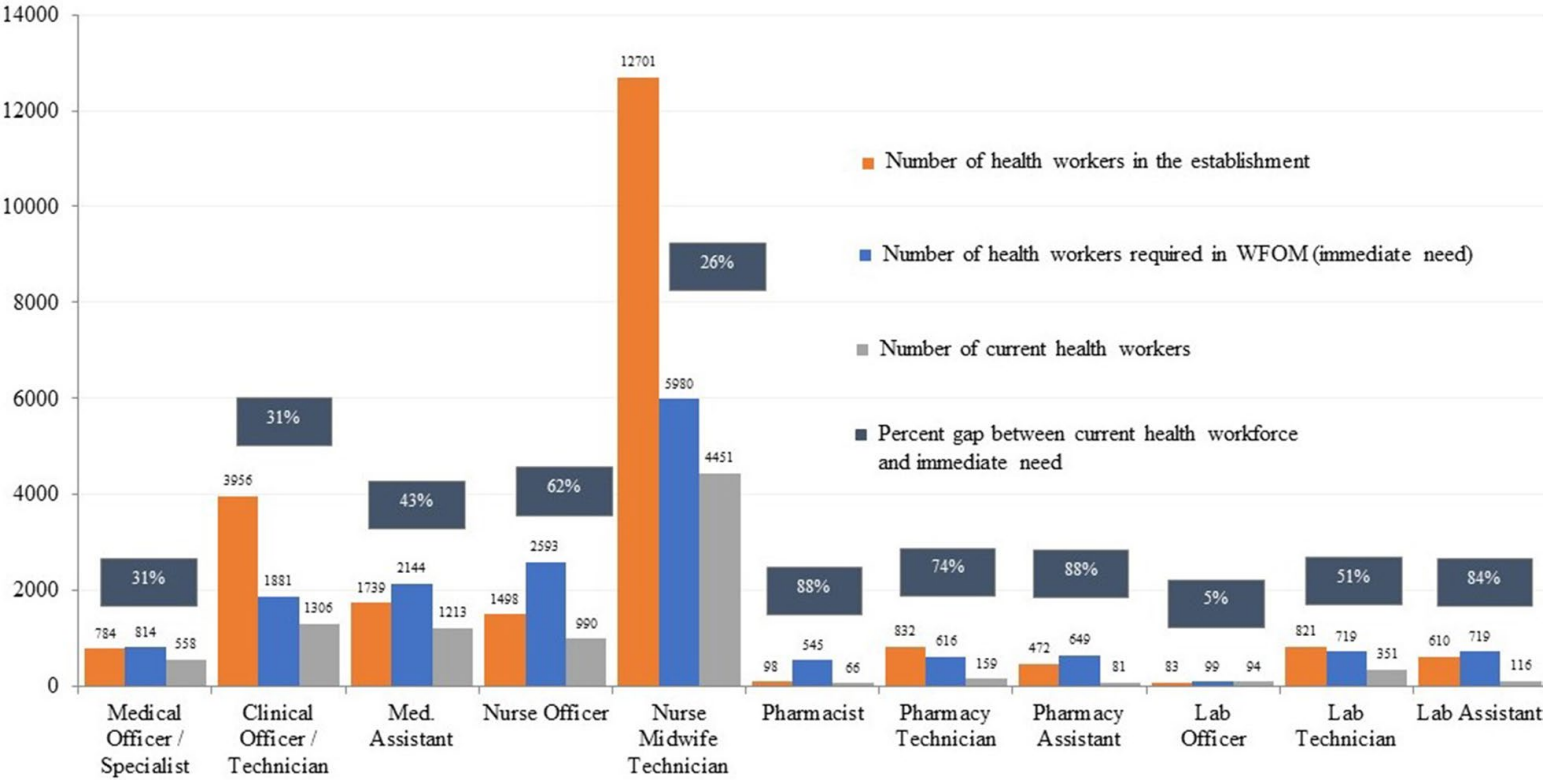
Current health workforce, optimized workforce, and establishment targets

The WFOM model results shown in Fig. 4 highlight the immediate need for health workers to optimally meet 2016 service utilization levels. The model shows a gap of 7374 health workers to optimally deliver services at current utilization levels, and a gap of 14 209 health workers between current staffing and long-term establishment targets for cadres included in the model. In absolute numbers, the largest gap between current health workers and immediate requirements is among nursing and midwifery officers, where the model recommends 1603 additional nurses and midwives to meet current levels of service utilization, a gap of 62%. The largest percentage gap is between the current 66 pharmacists and the immediate requirement of 545 pharmacists to meet current service utilization levels, a gap of 88% (479 pharmacists). In general, the WFOM results are lower than the establishment, as they represent the prioritized immediate need to meet current levels of service utilization. However, for several higher skilled cadres, such as medical officers, nursing and midwifery officers, pharmacists, and laboratory officers, the model estimates a greater immediate need than the total number required in the establishment.

**Fig. 5:**
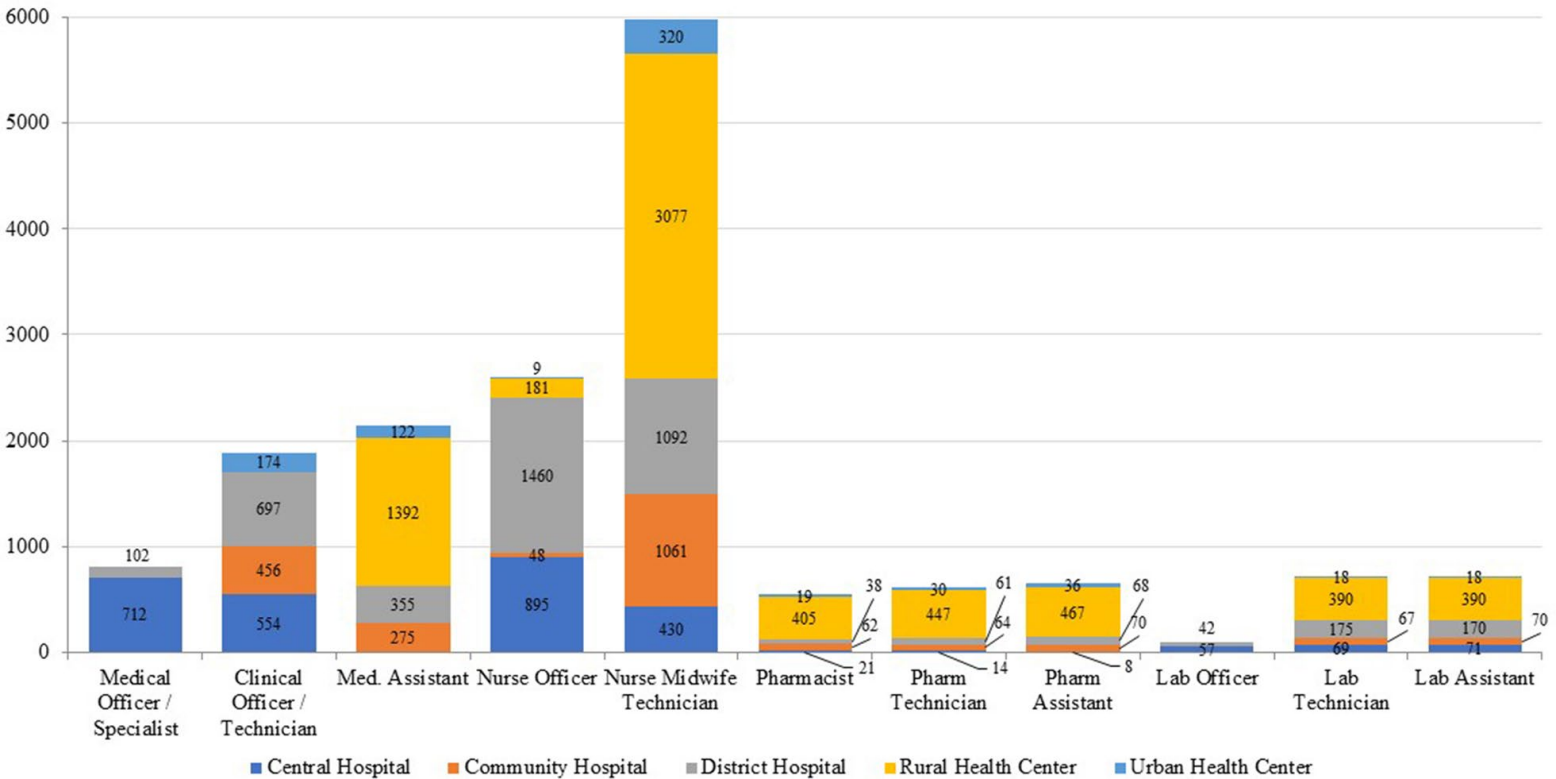
WFOM results on optimal health workforce by cadre and facility type

Optimized staffing levels to meet current service utilization by cadre and facility type are shown in Fig. 5. Nurse midwife technicians (NMTs) are the cadre with the highest staffing requirement as per the WFOM. The WFOM estimates a need for 5980 NMTs, of which 320 are needed in urban health centers, 3077 in rural health centers, 1092 in district hospitals, 1061 in community hospitals, and 430 in central hospitals.

Table 4 presents the results of the optimization analysis by district. It shows the percentage of posts to optimally deliver services at current utilization levels by cadre and district that are filled. There is wide variation by cadre and district in the proportion of positions that are filled, with the highest overall district vacancies in pharmacy and lab cadres. The WFOM highlights significant shortages for pharmacists in all districts, despite a high concentration in central hospitals. There is substantial variation in the concentration of nursing and midwifery officers across districts, with four districts showing posts filled at over 200% of optimized staffing levels, whereas 14 districts and two central hospitals have below 30% of their optimized need met.

**Table 4:**
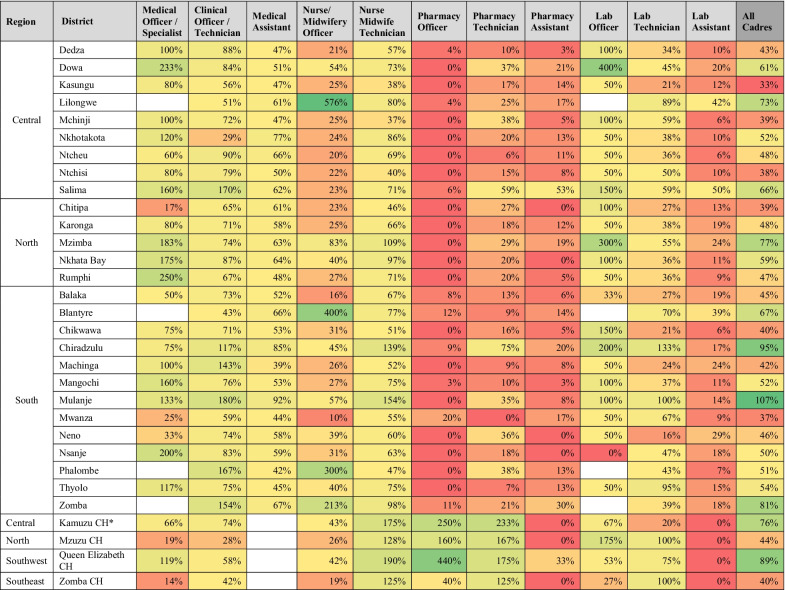
Percentage of optimized posts that are filled by district and cadre

*CH Central Hospital

## Discussion

Evidence must be carefully considered in designing a national HRH strategic plan to prioritize finite resources and develop actionable strategies reflective of the country context. The Malawi MoH and collaborators undertook an evidence-based approach to its HRH strategic planning process, developing a targeted data collection and analysis plan, and using vacancy analysis, workforce modelling, and scenario analysis to inform strategies recommended in the HRH SP 2018–2022. The results of these analyses are contained in the HRH SP, and the outputs were used to inform prioritization of objectives and interventions recommended in the strategic plan [26]. By highlighting data gaps and incorporating evidence throughout the HRH SP, the plan makes a strong case for strengthening HRH data systems and routinely using HRH data for planning and management of the health workforce [27].

The non-intervention scenario in the pipeline model highlights that if current trends in training, recruitment and attrition continue, Malawi will not meet its establishment within the strategic planning period. The intervention-based scenario analysis shows the most impactful intervention to close workforce gaps is doubling training enrollments. Malawi has already made significant investment in decentralizing education programs and scaling up rural health training, which are highly effective strategies to increase the rural health workforce [28﻿]. Further increasing training enrollments would require significant financial investment in infrastructure, student scholarships, and educators. Otherwise, quality of education may be compromised, a challenge which was previously observed in the rapid scale up of training during the EHRP period [2]. Furthermore, as the main employer of health workers in Malawi, MoH would need to prepare to recruit larger cohorts upon graduation, which would require advanced multi-sectoral and multi-year fiscal planning. Recognizing these are significant constraints in Malawi, where the country is already under pressure to reduce its wage bill [29], it would be unlikely that the government could sponsor large new enrollments and recruitments. Therefore, in the short term, the HRH SP recommends aligning training plans with recruitment needs to prioritize training to fill immediate workforce gaps, and strengthening data systems to better monitor and plan for new graduates to ensure they are hired in a timely manner.

The pipeline model illustrates that reducing voluntary attrition to 1% would allow Malawi to meet its establishment target by 2028 and forms the foundation for recommendations in the HRH SP to develop a retention strategy and strengthen health workforce management interventions. Significant research in Malawi has highlighted financial and non-financial strategies to improve retention which could be considered by government in developing a national retention strategy [30–33]. Given financial constraints in Malawi’s health sector, non-financial retention incentives such as housing provision, improved facility quality, and supervision would be more feasible to implement, as compared to financial incentives, such as increased salary or hazard pay. Importantly, in none of the intervention scenarios would Malawi meet the WHO recommendation of 4.45 health workers per 1000 population by 2030. This highlights the need for greater investment in the health workforce, in addition to efforts to optimize the existing workforce and align education plans with employment needs to more efficiently utilize existing resources.

The vacancy analysis highlights a workforce gap of 45% against national targets, with significant variation across districts. While this gap may seem intractable in Malawi’s fiscally constrained landscape [34], the WFOM provides a prioritized immediate term scenario that is actionable within the strategic planning period. For example, while NMTs form the backbone of Malawi’s primary care delivery system and, therefore, have the highest number of established posts and the greatest vacancy rate, the WFOM results prioritize the immediate recruitment of 1529 NMTs to meet current service utilization levels. This is a more realistic goal for the strategic plan period as compared to the gap of 8250 NMTs needed to meet the establishment. Using a demand-based approach to modeling human resource requirements, the WFOM results provide government with a tool to develop annual recruitment plans that prioritize districts and cadres with greatest need, and consider more efficient deployment of existing health workers. The model also provides government with a method to consider for reviewing national establishment targets based on current and projected service demand at facilities.

While the WFOM results generally recommend targets lower than the establishment for immediate term recruitment, for certain higher skilled frontline workers, such as medical and nursing officers, pharmacists, and lab officers, the WFOM results recommend recruitment of more health workers than the establishment. These results emphasize the importance of striving for a comprehensive and balanced mix of primary health care cadres within a national HRH strategy. Ensuring sufficient primary care workers with focus in medicine, nursing, pharmacy, and lab is critical to providing effective prevention and health promotion alongside continuous and coordinated care at the primary care level [28].

## Limitations

Data incompleteness and non-availability, and potential underreporting of service delivery data, impacted the scope of our analyses and required us to make assumptions, where data were not available (see Additional file 1). For example, duty station was not recorded in most staff return data, and therefore, we were unable to conduct vacancy and WFOM analyses at facility level. Furthermore, incomplete service delivery data led to exclusion of certain facilities from the analyses, and lack of data for services such as nutrition, mental health, and radiography led to the exclusion of certain cadres which provide these services from the WFOM. Recognizing these data gaps, the HRH SP makes a strong call to strengthen HRH and sector-wide data systems.

The judgment of clinical experts and observations of patient–provider interactions was used to determine the time it takes to deliver specific health services in the WFOM. While this is a widely accepted methodology for demand-based workforce modelling [8], clinical management of cases can vary significantly between providers, and clinical experts may have differing opinions on the ideal amount of time a service should take. To limit potential observation bias in the collection of activity times, multiple observations of the same activity were captured within and across facilities, and clinical experts were consulted to further validate the time estimates that were used as normative inputs for the WFOM model (see Additional file 1).

The models do not consider the service needs or production targets for cadres with advanced training required to deliver specialty services at the secondary and tertiary-level, as many of these training programs do not yet exist in Malawi. While the HRH SP makes recommendations regarding specialist training, additional work would be needed to validate these targets and to design models that could effectively project specialist needs. DCSAs are also excluded from the WFOM given their work takes place primarily outside the facility—at the community level. The DCSA targets in the HRH SP are drawn from the 2018 National Community Health Strategy [35], highlighting the importance of integrated sub-sectoral planning. Finally, as service utilization levels fall below national targets for service coverage articulated in the Health Sector Strategic Plan II 2017–2022 [4], the WFOM outputs should be considered as intermediate targets to provide high-quality services at current levels of utilization. However, these targets should be re-evaluated as utilization of services increases overtime.

The WFOM does not model scenarios that the government has yet to consider, such as productivity improvement in service delivery due to use of technology, task-shifting, or improved skill levels of health workers overtime. This is a common limitation in the use of workforce modeling for long-term planning [36]. However, as the WFOM is a flexible tool, it can be updated to incorporate any new scenarios overtime.

## Conclusions

Malawi’s HRH SP 2018–2022 presents a detailed vacancy analysis, HRH modeling, and scenario analysis to inform the strategies and interventions recommended in the plan, and makes a call to strengthen HRH data systems for improved workforce decision-making. The models and results presented in the HRH SP have been subsequently used to prioritize and cost HRH investments in national funding requests, to harmonize HRH projections across sub-sectoral plans, to catalyze momentum around HRH policy change, and have been incorporated into national and subnational annual planning tools and processes [37–40]. By following a practical methodology to identify information gaps, design data collection and analysis plans around these gaps, and utilize the evidence generated to build strategic priorities, Malawi’s HRH SP offers a replicable model that can be used in-country and beyond to promote evidence-informed strategic planning.

## Supplementary Information

**Additional file 1. **“Supplemental methodology for the pipeline model and Workforce Optimization Model,” this file provides supplemental information on the methodology, data sources, and assumptions used in the WFOM and pipeline models.

## Data Availability

The data sets used and/or analyzed during the current study are available from the corresponding author on reasonable request.

## Abbreviations

CHAI: Clinton Health Access Initiative
CHAM: Christian Health Association of Malawi
DCSA: Disease Control and Surveillance Assistant
DHIS2: District Health Information Software
EHRP: Emergency Human Resources Programme
HRH: Human resources for health
HRHSP: Human Resources for Health Strategic Plan
LMICs: Low- and middle-income countries
MoH: Ministry of Health
NMT: Nurse midwife technician
WISN: Workload Indicators of Staffing Need
WFOM: Workforce Optimization Model
