# Supplementary material for: Using modeling and scenario analysis to support evidence-based health workforce strategic planning in Malawi
Source: Hum Resour Health. 2022 Apr 18;20:34. doi: 10.1186/s12960-022-00730-3 (PMC9014573; doi:10.1186/s12960-022-00730-3)
Supplement: Supplementary file 1 — Additional file 1. “Supplemental methodology for the pipeline model and Workforce Optimization Model,” this file provides supplemental information on the methodology, data sources, and assumptions used in the WFOM and pipeline models. [file 12960_2022_730_MOESM1_ESM.docx]

**Additional file 1: supplemental methodology for the pipeline model and Workforce Optimization Model**

This file provides supplemental information on the methodology, data sources, and assumptions used in the pipeline model and Workforce Optimization Model (WFOM).

**Supplemental information on the pipeline model methodology and assumptions**

Data on student enrollments and the number of students graduating from 2012-2017 by cadre was collected at all training institutions in Malawi in September and October 2017 and validated in January and February 2018. Data on student enrollments was used to calculate future inflow of students into training institutions. Where data was not available from a training institution for a program, an average enrollment was calculated based on training programs of the same type at other schools. For the non-intervention scenario it was assumed that training enrollment would continue according to trends, expect for Disease Control and Surveillance Assistants (DCSAs), where training would increase according to the National Community Health Strategy, which indicates a plan to train at least 7,000 DCSAs by 2022 [1].

Using data on student enrollment and graduation, graduation rates were calculated for each training program. Graduation rates ranged from 75-100%, and for the baseline scenario, it was assumed that graduation rates would continue according to current trends by cadre. For cadres with licensing exam requirements, exam pass rate data was provided by the Nurses and Midwives Council of Malawi in October 2017, specifically, nursing officers (94.7%), nurse midwife technicians (95.1%), community midwifery assistants (91.1%), and psychiatric nursing officers (100%). DCSAs did not have a licensing exam at the time the analysis was conducted, but an exam was planned to be introduced, and 95% of graduates were assumed to pass. Other cadres do not have licensing exams, and therefore 100% of graduated students were assumed eligible for hire.

Hiring rates were calculated by comparing data from the Ministry of Health (MoH) and Christian Health Association of Malawi (CHAM) on number of new hires in 2015 and 2016 against training college data on number of new graduates. In 2015 and 2016 across all cadres the average absorption rate was approximately 50% and this was assumed for all future years and cadres, with the exception of DCSAs, who were assumed to have 100% absorption as they are specifically hired and trained to fill vacant posts.

The district staff return data includes information about place of birth, and this was used to develop assumptions about number of health workers hired from abroad. For cadres with no current training program in Malawi, recruitment of individuals trained outside the country was assumed to continue at similar rates as in previous years. The health worker cadres, programs, and training institutions included in the pipeline model are shared in Additional Tables 1 and 2 below.

Reliable and complete data on health worker attrition was not available to calculate health worker outflow. Through comparison of MoH analyses, analysis conducted in Malawi and other low-income countries (LICs) which suggested attrition of 14% for nurses and 15% for doctors, and MoH and stakeholder consultation, a 7% attrition rate for the non-intervention scenario was selected [2]. This includes 1% from retirement based on birthdate data in staff returns, 2% involuntary attrition, 3% voluntary attrition, and 1% study leave.

**Supplemental information on WFOM methodology and assumptions**

The package of services included in the Workforce Optimization Model (WFOM) was defined based on Malawi’s essential health package outlined in the Health Sector Strategic Plan II, taking into account data availability [3]. The services included in the model are shown in Additional Table 3 below.

Data for health worker activity times were collected for a prior iteration of the WFOM in 2010 [4]. Over 500 facility-based observations of provider-patient encounters timed with a stopwatch were conducted across a sample of 20 health facilities (including four central hospitals, four district hospitals, seven community or rural hospitals, and five health centers). In November 2017, MoH organized an expert consultation, during which these time-motion observations were compared with activity standard times collected from health worker interviews in 75 facilities during a 2017 Workload Indicators of Staffing Need (WISN) exercise [5]. Clinical experts then made adjustments for precision, as it is often the case that health workers see patients for shorter visits than recommended due to high patient volume. To accurately project the required workforce to deliver health services in high quality, the WFOM should utilize data on the recommended amount of time required for each service. Additional Tables 4 and 5 present the final health worker activity times included in the model.

The number of working hours available per year for each type of health worker was derived by subtracting the number of public holidays, vacation days, casual leave, sick days, maternity days (for female health workers only), and study leave from the number of workdays per year. The number of leave days was confirmed with MoH staff. Within each working day, health workers spend some time on activities that are not related to direct patient care, such as meetings, supply orders, teaching, general administrative tasks, and training. The expert consultation group convened by MoH in November 2017 reviewed the average amount of time spent per day on non-patient-facing activities per cadre included in the 2017 WISN Report [5]. The group then made adjustments based on clinical experience. The patient facing time per health worker cadre is shown in Additional Table 6.

**Additional Table 1. Health worker cadres and programs included in the pipeline model**

| **General Cadre** | **Position After Finishing Training** | **Training Programme Name** | **Academic**  **Award** | **Duration**  **(Years)** | **Upgrade Program**  **for Practicing**  **Health Workers** |
| --- | --- | --- | --- | --- | --- |
| Clinical | Medical Officer | MBBS | Degree | 6.0 | No |
| Clinical | Clinical Officer /Technician | Clinical Medicine (Dip-Upgrade) | Diploma | 1.5 | Yes |
| Clinical | Clinical Officer /Technician | Clinical Medicine (Dip) | Diploma | 3.0 | No |
| Clinical | Clinical Officer /Technician | Anesthesia (Dip-Upgrade) | Diploma | 1.5 | Yes |
| Clinical | Clinical Officer /Technician | Ear, Nose, Throat and Clinical Audiology (Dip-Upgrade) | Diploma | 1.5 | Yes |
| Clinical | Clinical Technician | Orthopedic Clinical Medicine (Dip) | Diploma | 3.0 | No |
| Clinical | Medical Assistant | Clinical Medicine (Cert) | Certificate | 2.0 | No |
| Dental | Dental Therapist | Dental Therapy (Dip) | Diploma | 3.0 | No |
| Environmental Health | Disease Control and Surveillance Assistant | Disease Control and Surveillance Assistant^[[1]](#footnote-1)^ | Training | 1.0 | No |
| Laboratory | Laboratory Technologist | Biomedical Sciences (BSc) | Degree | 5.0 | No |
| Laboratory | Laboratory Technician | Biomedical Sciences (Dip) | Diploma | 3.0 | No |
| Laboratory | Laboratory Assistant | Biomedical Sciences (Cert) | Certificate | 2.0 | No |
| Mental Health | Clinical Officer/ Mental Health | Clinical Medicine - Mental Health (BSc) | Degree | 4.0 | No |
| Mental Health | Nursing Officer/ Mental Health | Nursing - Mental Health and Psychiatric (BSc) | Degree | 4.0 | No |
| Mental Health | Nursing Technician/ Mental Health | Nursing - Mental Health and Psychiatric (Dip - Upgrade) | Diploma | 1.0 | Yes |
| Mental Health | Psychosocial Counsellor | Psychosocial Counselling (Dip) | Diploma | 3.0 | No |
| Mental Health | Psychiatric Nursing Officer | Nursing - Mental Health and Psychiatric (BSc - Upgrade) | Degree | 3.0 | Yes |
| Nursing | Nursing Officer | Midwifery (BSc) | Degree | 4.0 | No |
| Nursing | Nursing Officer | Nursing - Adult Health (BSc) | Degree | 4.0 | No |
| Nursing | Nursing Officer | Nursing - Child Health (BSc) | Degree | 4.0 | No |
| Nursing | Nursing Officer | Nursing and Midwifery (BSc) | Degree | 4.0 | No |
| Nursing | Nursing Officer | Community Health Nursing (BSc) | Degree | 4.0 | No |
| Nursing | Nursing Officer | Nursing - Adult Health (BSc-Upgrade) | Degree | 3.0 | Yes |
| Nursing | Nursing Officer | Nursing - Child Health (BSc-Upgrade) | Degree | 3.0 | Yes |
| Nursing | Nursing Officer | Community Health Nursing (BSc-Upgrade) | Degree | 3.0 | Yes |
| Nursing | Nursing Officer | Midwifery (BSc- Upgrade) | Degree | 3.0 | Yes |
| Nursing | Nurse Midwife Technician | Community Health Nursing (Dip - Upgrade) | Diploma | 1.0 | Yes |
| Nursing | Nurse Midwife Technician | Registered Nursing and Midwifery (Dip) | Diploma | 3.0 | No |
| Nursing | Nurse Midwife Technician | Nurse Midwife Technician (Dip) | Diploma | 3.0 | No |
| Nursing | Nurse Midwife Technician | Registered Nursing (Dip) | Diploma | 3.0 | No |
| Nursing | Nurse Midwife Technician | Registered Nursing and Midwifery (Dip - Upgrade) | Diploma | 2.0 | Yes |
| Nursing | Community Midwifery Assistant | Community Midwifery (Cert) | Certificate | 1.5 | No |
| Nutrition | Nutritionist | Food Science and Nutrition (BSc) | Degree | 4.0 | No |
| Pharmacy | Pharmacist | Pharmacy (BSc) | Degree | 5.0 | No |
| Pharmacy | Pharmacy Technician | Pharmacy (Dip) | Diploma | 3.0 | No |
| Pharmacy | Pharmacy Assistant | Pharmacy (Cert) | Certificate | 2.0 | No |
| Radiography | Radiography Technician | Radiography (Dip) | Diploma | 3.0 | No |
| Specialists | Optometrist | Optometry (BSc) | Degree | 5.0 | No |
| Specialists | Physiotherapist | Physiotherapy (BSc) | Degree | 5.0 | No |
| Specialists | Optometry Technician | Optometry (Dip) | Diploma | 3.0 | No |
| Specialists | Clinical Technician | Ophthalmology, Cataract Surgery (Dip-Upgrade) | Diploma | 1.5 | **Yes** |
| Specialists | Clinical Technician | Ophthalmology, General (Dip-Upgrade) | Diploma | 1.5 | **Yes** |
| Specialists | Clinical Technician | Orthopedics (Dip-Upgrade) | Diploma | 1.5 | **Yes** |

**Additional Table 2. Health worker training institutions and programs included in the pipeline model**

| **Institution** | **Programmes** |
| --- | --- |
| **Catholic University of Malawi** | Nursing and Midwifery (BSc) |
| **Daeyang College of Nursing** | Nursing and Midwifery (BSc) |
|  | Nurse Midwife Technician (Dip) |
| **Ekwendeni College of Health Sciences** | Clinical Medicine (Dip) |
|  | Community Midwifery (Cert) |
|  | Nurse Midwife Technician (Dip) |
|  | Registered Nursing (Dip) |
| **Holy Family College** | Community Midwifery (Cert) |
|  | Nurse Midwife Technician (Dip) |
| **Lilongwe University of Agriculture and Natural Resources** | Food Science and Nutrition (BSc) |
| **Malamulo College of Health Sciences** | Clinical Medicine (Dip) |
|  | Clinical Medicine (Cert) |
|  | Biomedical Sciences (BSc) |
|  | Biomedical Sciences (Dip) |
|  | Nurse Midwife Technician (Dip) |
| **Malawi College of Health Sciences – Blantyre** | Anaesthesia (Dip-Upgrade) |
|  | Clinical Medicine (Dip-Upgrade) |
|  | Ear, Nose, Throat and Clinical Audiology (Dip-Upgrade) |
|  | Clinical Medicine (Cert) |
|  | Community Midwifery (Cert) |
|  | Registered Nursing and Midwifery (Dip) |
|  | Registered Nursing (Dip) |
|  | Orthopaedic Clinical Medicine (Dip) |
|  | Orthopadics (Dip-Upgrade) |
| **Malawi College of Health Sciences – Lilongwe** | Anaesthesia (Dip-Upgrade) |
|  | Clinical Medicine (Dip) |
|  | Clinical Medicine (Cert) |
|  | Dental Therapy (Dip) |
|  | Biomedical Sciences (Dip) |
|  | Biomedical Sciences (Cert) |
|  | Community Health Nursing (Dip - Upgrade) |
|  | Nurse Midwife Technician (Dip) |
|  | Pharmacy (Dip) |
|  | Pharmacy (Cert) |
|  | Radiography (Dip) |
|  | Optometry (Dip) |
|  | Ophthamology, Cataract Surgery (Dip-Upgrade) |
|  | Ophthamology, General (Dip-Upgrade) |
| **Malawi College of Health Sciences – Zomba** | Nursing - Mental Health and Psychiatric (Dip - Upgrade) |
|  | Community Midwifery (Cert) |
|  | Nurse Midwife Technician (Dip) |
| **MoH DCSA Training** | Disease Control and Surveillance Assistant |
| **Mulanje Mission College** | Community Midwifery (Cert) |
|  | Nurse Midwife Technician (Dip) |
| **Mzuzu University** | Biomedical Sciences (BSc) |
|  | Nursing and Midwifery (BSc) |
|  | Optometry (BSc) |
| **Nkhoma College of Nursing and Midwifery** | Community Midwifery (Cert) |
|  | Nurse Midwife Technician (Dip) |
| **St. John of God College of Health Sciences College** | Clinical Medicine - Mental Health (BSc) |
|  | Nursing - Mental Health and Psychiatry (BSc) |
|  | Psychosocial Counselling (Dip) |
|  | Nurse Midwife Technician (Dip) |
| **St. John's College of Nursing/Midwifery** | Community Midwifery (Cert) |
|  | Nurse Midwife Technician (Dip) |
| **St. Joseph College of Nursing** | Community Midwifery (Cert) |
|  | Nurse Midwife Technician (Dip) |
| **St. Luke’s College of Nursing** | Community Midwifery (Cert) |
|  | Nurse Midwife Technician (Dip) |
| **Trinity College of Nursing and Midwifery** | Community Midwifery (Cert) |
|  | Nurse Midwife Technician (Dip) |
| **University of Malawi - Chancellor College** | Food Science and Nutrition (BSc) |
| **University of Malawi - College of Medicine** | MBBS |
|  | Biomedical Sciences (BSc) |
|  | Pharmacy (BSc) |
|  | Physiotherapy (BSc) |
| **University of Malawi - Kamuzu College of Nursing** | Nursing - Mental Health and Psychiatry (BSc) |
|  | Community Health Nursing (BSc) |
|  | Nursing and Midwifery (BSc) |
|  | Midwifery (BSc) |
|  | Nursing - Adult Health (BSc) |
|  | Nursing - Child Health (BSc) |
|  | Community Midwifery (Cert) |
|  | Nursing - Mental Health and Psychiatry (BSc - Upgrade) |
|  | Nursing - Adult Health (BSc-Upgrade) |
|  | Nursing - Child Health (BSc-Upgrade) |
|  | Community Health Nursing (BSc-Upgrade) |
|  | Midwifery (BSc-Upgrade) |

Additional Table 3. Services included in the WFOM by category

| **Service category** | **Service name** | **Service description** | **Data source** |
| --- | --- | --- | --- |
| **Inpatient / outpatient care** | Over-five outpatient department visits | Number of OPD visits for patients over five | HMIS |
|  | Under-five outpatient department visits | Number of OPD visits for patients under five | HMIS |
|  | Inpatient days | Total number of inpatient bed days | HMIS |
|  | Inpatient admission | Total number of unique patients admitted for inpatient stay (of any length) | HMIS |
| **HIV** | PMTCT visits | Number of ART visits for pregnant women newly on ART | Dept of HIV/AIDS data |
|  | Adult HIV treatment visit - established patient, stable | Number of ART visits for established, stable adult patients (not their first year) | Dept of HIV/AIDS data |
|  | Adult HIV treatment visit - new patient | Number of new ART initiations for adults (first year of treatment) | Dept of HIV/AIDS data |
|  | Adult HIV treatment visit - established patient, non- stable | Number of ART visits for established, non-stable adult patients (not their first year) | Dept of HIV/AIDS data |
|  | Pediatric HIV treatment visit | Number of ART visits for pediatric patients (new or established) | Dept of HIV/AIDS data |
|  | HCT Negative | Number of HIV tests provided, negative result | HMIS |
|  | HCT Positive | Number of HIV tests provided, positive result | HMIS |
|  | Male Circumcision | Number of male circ procedures completed | HMIS |
| **TB** | TB visit - New patient | Number of new patients initiated on TB treatment (first visit) | Dept of HIV/AIDS data |
|  | TB visit – Follow-up visit | Number of TB treatment visits for established patient (not first visit) | Dept of HIV/AIDS data |
| **RMNCH** | STI treatment | Number of STI treatment visits | Dept of HIV/AIDS data |
|  | ANC - first visit | Number of first ANC visits | HMIS |
|  | ANC - follow-up visit | Number of ANC visits for patients that have already been seen at least once in that pregnancy | HMIS |
|  | Caesarian Section | Number of c-section deliveries | HMIS |
|  | Complicated Delivery | Number of complicated deliveries | HMIS |
|  | Normal Delivery | Number of normal deliveries | HMIS |
|  | EPI visit | Number of EPI visits (each visit could include multiple immunization but 1 immunization indicator was used as a proxy for the visit: 6 wk visit- DTP1, 10 wk visit- DTP2, 14 wk visit- DTP3, 9 mo visit- Measles) | HMIS |
|  | Under-five malnutrition | Number of cases of severe U5 mal-nutrition | HMIS |
|  | Family planning visit | Number of family planning visits (to receive any type of FP service) -- should be for everybody but we could only get new client | HMIS |
| **Misc** | Accidents and Emergencies | Number of patients treated for injuries due to accidents or emergencies | HMIS |
|  | Major Surgery | Number of major surgeries | Data from several hospitals |
|  | Minor Surgery | Number of minor surgeries | Data from several hospitals |
| **Lab** | Histology | Histology | MoH lab data |
|  | Biochemistry | Blood glucose, bilirubin, urea, etc. | MoH lab data |
|  | Cytology | CD4 absolute count | MoH lab data |
|  | Haematology | Full blood count, WBC differential, CD4 percentage, etc. | MoH lab data |
|  | Microbiology | Cerebral spinal fluid, etc. | MoH lab data |
|  | Molecular | DNA-EID, viral load, etc. | MoH lab data |
|  | POC tests | Malaria rapid diagnostics tests | HMIS |
|  | Parasitology | Malaria microscopy, Urine chemistry, etc. | MoH lab data |
|  | Serology | Syphilis, pregnancy, etc. | MoH lab data |
|  | TB Microbiology | GeneXpert, sputum, etc. | MoH lab data |
|  | Transfusions | Blood transfusion testing | MoH lab data |

**Additional Table 4. Activity times by cadre and health facility type in the WFOM**

| **Activity** | **Cadre** | **Central Hospital** | |  | **District Hospital** | |  | **Community Hospital** | |  | **Urban Health Center** | |  | **Rural Health Center** | |
| --- | --- | --- | --- | --- | --- | --- | --- | --- | --- | --- | --- | --- | --- | --- | --- |
|  |  | **Minutes** | **Percentage^1^** |  | **Minutes** | **Percentage** |  | **Minutes** | **Percentage** |  | **Minutes** | **Percentage** |  | **Minutes** | **Percentage** |
| *Inpatient Admissions (Ongoing Monitoring)* | Medical Officer | **5.0** | **100%** |  | **5.0** | **25%** |  | 0.0 | 0% |  | 0.0 | 0% |  | 0.0 | 0% |
|  | Clinical Officer | **7.0** | **100%** |  | **7.0** | **100%** |  | **7.0** | **100%** |  | **7.0** | **100%** |  | 0.0 | 0% |
|  | Nursing Officer | **40.0** | **30%** |  | **30.0** | **30%** |  | 0.0 | 0% |  | 0.0 | 0% |  | 0.0 | 0% |
|  | Nurse Midwife Technician | **40.0** | **70%** |  | **30.0** | **70%** |  | **30.0** | **100%** |  | **20.0** | **100%** |  | **20.0** | **100%** |
|  | Medical Assistant | 0.0 | 0% |  | 0.0 | 0% |  | 0.0 | 0% |  | 0.0 | 0% |  | 0.0 | 0% |
|  | Pharmacist | **2.0** | **100%** |  | **2.0** | **100%** |  | **2.0** | **100%** |  | 0.0 | 0% |  | 0.0 | 0% |
|  | Pharmacy Technician | 0.0 | 0% |  | 0.0 | 0% |  | 0.0 | 0% |  | **2.0** | **50%** |  | **2.0** | **50%** |
|  | Pharmacy Assistant | 0.0 | 0% |  | 0.0 | 0% |  | 0.0 | 0% |  | **2.0** | **50%** |  | **2.0** | **50%** |
|  |  |  |  |  |  |  |  |  |  |  |  |  |  |  |  |
| *Inpatient Admissions and Discharge Process* | Medical Officer | **15.0** | **50%** |  | 0.0 | 0% |  | 0.0 | 0% |  | 0.0 | 0% |  | 0.0 | 0% |
|  | Clinical Officer | **20.0** | **50%** |  | **20.0** | **50%** |  | **20.0** | **50%** |  | **20.0** | **40%** |  | 0.0 | 0% |
|  | Nursing Officer | **20.0** | **30%** |  | **20.0** | **30%** |  | 0.0 | 0% |  | 0.0 | 0% |  | 0.0 | 0% |
|  | Nurse Midwife Technician | **20.0** | **70%** |  | **20.0** | **70%** |  | **20.0** | **100%** |  | **20.0** | **100%** |  | **20.0** | **100%** |
|  | Medical Assistant | 0.0 | 0% |  | **15.0** | **50%** |  | **15.0** | **50%** |  | **15.0** | **40%** |  | **15.0** | **30%** |
|  | Pharmacist | 0.0 | 0% |  | 0.0 | 0% |  | 0.0 | 0% |  | 0.0 | 0% |  | 0.0 | 0% |
|  | Pharmacy Technician | 0.0 | 0% |  | 0.0 | 0% |  | 0.0 | 0% |  | 0.0 | 0% |  | 0.0 | 0% |
|  | Pharmacy Assistant | **2.0** | **100%** |  | **2.0** | **100%** |  | **2.0** | **100%** |  | **2.0** | **100%** |  | **2.0** | **50%** |
|  |  |  |  |  |  |  |  |  |  |  |  |  |  |  |  |
| *Under 5 Outpatient Visit* | Medical Officer | **10.0** | **50%** |  | 0.0 | 0% |  | 0.0 | 0% |  | 0.0 | 0% |  | 0.0 | 0% |
|  | Clinical Officer | **12.0** | **50%** |  | **12.0** | **50%** |  | **12.0** | **50%** |  | **12.0** | **50%** |  | 0.0 | 0% |
|  | Nursing Officer | **7.0** | **50%** |  | **7.0** | **50%** |  | 0.0 | 0% |  | 0.0 | 0% |  | 0.0 | 0% |
|  | Nurse Midwife Technician | **7.0** | **50%** |  | **7.0** | **100%** |  | **7.0** | **100%** |  | **7.0** | **100%** |  | **7.0** | **100%** |
|  | Medical Assistant | 0.0 | 0% |  | **10.0** | **50%** |  | **10.0** | **50%** |  | **10.0** | **50%** |  | **10.0** | **50%** |
|  | Pharmacist | 0.0 | 0% |  | 0.0 | 0% |  | 0.0 | 0% |  | 0.0 | 0% |  | 0.0 | 0% |
|  | Pharmacy Technician | **2.0** | **40%** |  | **2.0** | **40%** |  | **2.0** | **40%** |  | **2.0** | **40%** |  | **2.0** | **40%** |
|  | Pharmacy Assistant | **2.0** | **40%** |  | **2.0** | **40%** |  | **2.0** | **40%** |  | **2.0** | **40%** |  | **2.0** | **40%** |
|  |  |  |  |  |  |  |  |  |  |  |  |  |  |  |  |
| *Over 5 Outpatient visit* | Medical Officer | **7.0** | **50%** |  | 0.0 | 0% |  | 0.0 | 0% |  | 0.0 | 0% |  | 0.0 | 0% |
|  | Clinical Officer | **12.0** | **50%** |  | **12.0** | **50%** |  | **12.0** | **50%** |  | **12.0** | **50%** |  | 0.0 | 0% |
|  | Nursing Officer | **5.0** | **20%** |  | **5.0** | **20%** |  | 0.0 | 0% |  | 0.0 | 0% |  | 0.0 | 0% |
|  | Nurse Midwife Technician | **5.0** | **80%** |  | **5.0** | **80%** |  | **5.0** | **100%** |  | **5.0** | **100%** |  | **5.0** | **100%** |
|  | Medical Assistant | 0.0 | 0% |  | **7.0** | **50%** |  | **7.0** | **50%** |  | **7.0** | **50%** |  | **7.0** | **50%** |
|  | Pharmacist | 0.0 | 0% |  | 0.0 | 0% |  | 0.0 | 0% |  | 0.0 | 0% |  | 0.0 | 0% |
|  | Pharmacy Technician | 0.0 | 0% |  | **2.0** | **40%** |  | **2.0** | **40%** |  | **2.0** | **40%** |  | **2.0** | **40%** |
|  | Pharmacy Assistant | 0.0 | 0% |  | **2.0** | **40%** |  | **2.0** | **40%** |  | **2.0** | **40%** |  | **2.0** | **40%** |
|  |  |  |  |  |  |  |  |  |  |  |  |  |  |  |  |
| *Normal Deliveries* | Medical Officer | 0.0 | 0% |  | 0.0 | 0% |  | 0.0 | 0% |  | 0.0 | 0% |  | 0.0 | 0% |
|  | Clinical Officer | 0.0 | 0% |  | 0.0 | 0% |  | 0.0 | 0% |  | 0.0 | 0% |  | 0.0 | 0% |
|  | Nursing Officer | **118.0** | **45%** |  | **118.0** | **45%** |  | 0.0 | 0% |  | 0.0 | 0% |  | 0.0 | 0% |
|  | Nurse Midwife Technician | **118.0** | **70%** |  | **118.0** | **70%** |  | **118.0** | **100%** |  | **118.0** | **100%** |  | **118.0** | **100%** |
|  | Medical Assistant | 0.0 | 0% |  | 0.0 | 0% |  | 0.0 | 0% |  | 0.0 | 0% |  | **45.0** | **30%** |
|  | Pharmacist | 0.0 | 0% |  | 0.0 | 0% |  | 0.0 | 0% |  | 0.0 | 0% |  | 0.0 | 0% |
|  | Pharmacy Technician | **2.0** | **100%** |  | **2.0** | **50%** |  | **2.0** | **50%** |  | **2.0** | **50%** |  | **2.0** | **50%** |
|  | Pharmacy Assistant | 0.0 | 0% |  | **2.0** | **50%** |  | **2.0** | **50%** |  | **2.0** | **50%** |  | **2.0** | **50%** |
|  |  |  |  |  |  |  |  |  |  |  |  |  |  |  |  |
| *Complicated Deliveries* | Medical Officer | **30.0** | **35%** |  | **30.0** | **10%** |  | 0.0 | 0% |  | 0.0 | 0% |  | 0.0 | 0% |
|  | Clinical Officer | **30.0** | **65%** |  | **30.0** | **70%** |  | **30.0** | **100%** |  | **30.0** | **100%** |  | 0.0 | 0% |
|  | Nursing Officer | **30.0** | **100%** |  | **30.0** | **100%** |  | 0.0 | 0% |  | 0.0 | 0% |  | 0.0 | 0% |
|  | Nurse Midwife Technician | **30.0** | **100%** |  | **30.0** | **100%** |  | **30.0** | **100%** |  | **30.0** | **100%** |  | **30.0** | **100%** |
|  | Medical Assistant | 0.0 | 0% |  | 0.0 | 0% |  | 0.0 | 0% |  | **5.0** | **50%** |  | **5.0** | **50%** |
|  | Pharmacist | 0.0 | 0% |  | 0.0 | 0% |  | 0.0 | 0% |  | 0.0 | 0% |  | 0.0 | 0% |
|  | Pharmacy Technician | **2.0** | **100%** |  | **2.0** | **50%** |  | **2.0** | **50%** |  | **2.0** | **50%** |  | **2.0** | **50%** |
|  | Pharmacy Assistant | 0.0 | 0% |  | **2.0** | **50%** |  | **2.0** | **50%** |  | **2.0** | **50%** |  | **2.0** | **50%** |
|  |  |  |  |  |  |  |  |  |  |  |  |  |  |  |  |
| *Caesarean Sections* | Medical Officer | **45.0** | **50%** |  | **45.0** | **20%** |  | 0.0 | 0% |  | 0.0 | 0% |  | 0.0 | 0% |
|  | Clinical Officer | **45.0** | **50%** |  | **45.0** | **80%** |  | **45.0** | **100%** |  | **45.0** | **100%** |  | 0.0 | 0% |
|  | Nursing Officer | **50.0** | **100%** |  | **50.0** | **100%** |  | 0.0 | 0% |  | 0.0 | 0% |  | 0.0 | 0% |
|  | Nurse Midwife Technician | **50.0** | **100%** |  | **50.0** | **100%** |  | **100.0** | **100%** |  | **100.0** | **100%** |  | 0.0 | 0% |
|  | Medical Assistant | 0.0 | 0% |  | 0.0 | 0% |  | 0.0 | 0% |  | 0.0 | 0% |  | 0.0 | 0% |
|  | Pharmacist | **10.0** | **50%** |  | 0.0 | 0% |  | 0.0 | 0% |  | 0.0 | 0% |  | 0.0 | 0% |
|  | Pharmacy Technician | **10.0** | **50%** |  | **10.0** | **100%** |  | **10.0** | **100%** |  | **10.0** | **100%** |  | 0.0 | 0% |
|  | Pharmacy Assistant | 0.0 | 0% |  | 0.0 | 0% |  | 0.0 | 0% |  | 0.0 | 0% |  | 0.0 | 0% |
|  |  |  |  |  |  |  |  |  |  |  |  |  |  |  |  |
| *Family Planning* | Medical Officer | 0.0 | 0% |  | 0.0 | 0% |  | 0.0 | 0% |  | 0.0 | 0% |  | 0.0 | 0% |
|  | Clinical Officer | 0.0 | 0% |  | 0.0 | 0% |  | 0.0 | 0% |  | 0.0 | 0% |  | 0.0 | 0% |
|  | Nursing Officer | **20.0** | **50%** |  | **20.0** | **50%** |  | 0.0 | 0% |  | 0.0 | 0% |  | 0.0 | 0% |
|  | Nurse Midwife Technician | **20.0** | **50%** |  | **20.0** | **50%** |  | **20.0** | **80%** |  | **20.0** | **80%** |  | **20.0** | **80%** |
|  | Medical Assistant | 0.0 | 0% |  | 0.0 | 0% |  | 0.0 | 0% |  | 0.0 | 0% |  | 0.0 | 0% |
|  | Pharmacist | 0.0 | 0% |  | 0.0 | 0% |  | 0.0 | 0% |  | 0.0 | 0% |  | 0.0 | 0% |
|  | Pharmacy Technician | 0.0 | 0% |  | 0.0 | 0% |  | 0.0 | 0% |  | 0.0 | 0% |  | 0.0 | 0% |
|  | Pharmacy Assistant | 0.0 | 0% |  | 0.0 | 0% |  | 0.0 | 0% |  | 0.0 | 0% |  | 0.0 | 0% |
|  |  |  |  |  |  |  |  |  |  |  |  |  |  |  |  |
| *Antenatal Care - First Visit* | Medical Officer | **15.0** | **10%** |  | 0.0 | 0% |  | 0.0 | 0% |  | 0.0 | 0% |  | 0.0 | 0% |
|  | Clinical Officer | **15.0** | **10%** |  | **15.0** | **20%** |  | **15.0** | **10%** |  | **15.0** | **10%** |  | 0.0 | 0% |
|  | Nursing Officer | **30.0** | **100%** |  | **30.0** | **100%** |  | 0.0 | 0% |  | 0.0 | 0% |  | 0.0 | 0% |
|  | Nurse Midwife Technician | **30.0** | **100%** |  | **30.0** | **100%** |  | **30.0** | **100%** |  | **30.0** | **100%** |  | **30.0** | **100%** |
|  | Medical Assistant | 0.0 | 0% |  | 0.0 | 0% |  | **15.0** | **10%** |  | **15.0** | **10%** |  | **15.0** | **20%** |
|  | Pharmacist | 0.0 | 0% |  | 0.0 | 0% |  | 0.0 | 0% |  | 0.0 | 0% |  | 0.0 | 0% |
|  | Pharmacy Technician | 0.0 | 0% |  | 0.0 | 0% |  | 0.0 | 0% |  | 0.0 | 0% |  | 0.0 | 0% |
|  | Pharmacy Assistant | 0.0 | 0% |  | 0.0 | 0% |  | 0.0 | 0% |  | 0.0 | 0% |  | 0.0 | 0% |
|  |  |  |  |  |  |  |  |  |  |  |  |  |  |  |  |
| *Antenatal Care - Followup Visit* | Medical Officer | **5.0** | **10%** |  | **5.0** | **5%** |  | 0.0 | 0% |  | 0.0 | 0% |  | 0.0 | 0% |
|  | Clinical Officer | **5.0** | **10%** |  | **5.0** | **15%** |  | **5.0** | **10%** |  | **5.0** | **10%** |  | 0.0 | 0% |
|  | Nursing Officer | 0.0 | 0% |  | 0.0 | 0% |  | 0.0 | 0% |  | 0.0 | 0% |  | 0.0 | 0% |
|  | Nurse Midwife Technician | **15.0** | **100%** |  | **15.0** | **100%** |  | **15.0** | **100%** |  | **15.0** | **100%** |  | **15.0** | **100%** |
|  | Medical Assistant | 0.0 | 0% |  | 0.0 | 0% |  | **5.0** | **10%** |  | **5.0** | **10%** |  | **5.0** | **20%** |
|  | Pharmacist | 0.0 | 0% |  | 0.0 | 0% |  | 0.0 | 0% |  | 0.0 | 0% |  | 0.0 | 0% |
|  | Pharmacy Technician | 0.0 | 0% |  | 0.0 | 0% |  | 0.0 | 0% |  | 0.0 | 0% |  | 0.0 | 0% |
|  | Pharmacy Assistant | 0.0 | 0% |  | 0.0 | 0% |  | 0.0 | 0% |  | 0.0 | 0% |  | 0.0 | 0% |
|  |  |  |  |  |  |  |  |  |  |  |  |  |  |  |  |
| *EPI* | Medical Officer | 0.0 | 0% |  | 0.0 | 0% |  | 0.0 | 0% |  | 0.0 | 0% |  | 0.0 | 0% |
|  | Clinical Officer | 0.0 | 0% |  | 0.0 | 0% |  | 0.0 | 0% |  | 0.0 | 0% |  | 0.0 | 0% |
|  | Nursing Officer | 0.0 | 0% |  | 0.0 | 0% |  | 0.0 | 0% |  | 0.0 | 0% |  | 0.0 | 0% |
|  | Nurse Midwife Technician | **5.0** | **20%** |  | **5.0** | **20%** |  | **5.0** | **20%** |  | **5.0** | **10%** |  | **5.0** | **10%** |
|  | Medical Assistant | 0.0 | 0% |  | 0.0 | 0% |  | 0.0 | 0% |  | 0.0 | 0% |  | 0.0 | 0% |
|  | Pharmacist | 0.0 | 0% |  | 0.0 | 0% |  | 0.0 | 0% |  | 0.0 | 0% |  | 0.0 | 0% |
|  | Pharmacy Technician | 0.0 | 0% |  | 0.0 | 0% |  | 0.0 | 0% |  | 0.0 | 0% |  | 0.0 | 0% |
|  | Pharmacy Assistant | **1.0** | **100%** |  | **1.0** | **100%** |  | **1.0** | **100%** |  | **1.0** | **100%** |  | **1.0** | **100%** |
|  |  |  |  |  |  |  |  |  |  |  |  |  |  |  |  |
| *STI* | Medical Officer | 0.0 | 0% |  | 0.0 | 0% |  | 0.0 | 0% |  | 0.0 | 0% |  | 0.0 | 0% |
|  | Clinical Officer | **5.0** | **100%** |  | **10.0** | **20%** |  | **10.0** | **20%** |  | **10.0** | **20%** |  | 0.0 | 0% |
|  | Nursing Officer | **20.0** | **25%** |  | **20.0** | **25%** |  | 0.0 | 0% |  | 0.0 | 0% |  | 0.0 | 0% |
|  | Nurse Midwife Technician | **20.0** | **25%** |  | **20.0** | **25%** |  | **20.0** | **50%** |  | **20.0** | **50%** |  | **20.0** | **50%** |
|  | Medical Assistant | 0.0 | 0% |  | **10.0** | **30%** |  | **10.0** | **30%** |  | **10.0** | **30%** |  | **10.0** | **50%** |
|  | Pharmacist | **2.0** | **10%** |  | **2.0** | **10%** |  | **2.0** | **10%** |  | **2.0** | **10%** |  | **2.0** | **10%** |
|  | Pharmacy Technician | **3.0** | **50%** |  | **3.0** | **50%** |  | 0.0 | 0% |  | 0.0 | 0% |  | 0.0 | 0% |
|  | Pharmacy Assistant | **3.0** | **50%** |  | **3.0** | **50%** |  | **3.0** | **50%** |  | **3.0** | **50%** |  | **3.0** | **50%** |
|  |  |  |  |  |  |  |  |  |  |  |  |  |  |  |  |
| *Growth Monitoring* | Medical Officer | 0.0 | 0% |  | 0.0 | 0% |  | 0.0 | 0% |  | 0.0 | 0% |  | 0.0 | 0% |
|  | Clinical Officer | 0.0 | 0% |  | **5.0** | **20%** |  | **5.0** | **20%** |  | 0.0 | 0% |  | 0.0 | 0% |
|  | Nursing Officer | 0.0 | 0% |  | 0.0 | 0% |  | 0.0 | 0% |  | 0.0 | 0% |  | 0.0 | 0% |
|  | Nurse Midwife Technician | 0.0 | 0% |  | 0.0 | 0% |  | **5.0** | **50%** |  | **5.0** | **100%** |  | **5.0** | **50%** |
|  | Medical Assistant | 0.0 | 0% |  | **5.0** | **20%** |  | **5.0** | **20%** |  | 0.0 | 0% |  | **5.0** | **20%** |
|  | Pharmacist | 0.0 | 0% |  | 0.0 | 0% |  | 0.0 | 0% |  | 0.0 | 0% |  | 0.0 | 0% |
|  | Pharmacy Technician | 0.0 | 0% |  | 0.0 | 0% |  | 0.0 | 0% |  | 0.0 | 0% |  | 0.0 | 0% |
|  | Pharmacy Assistant | 0.0 | 0% |  | 0.0 | 0% |  | 0.0 | 0% |  | 0.0 | 0% |  | 0.0 | 0% |
|  |  |  |  |  |  |  |  |  |  |  |  |  |  |  |  |
| *Treatment of U5 Severe Malnutirion* | Medical Officer | **5.0** | **20%** |  | **5.0** | **20%** |  | 0.0 | 0% |  | 0.0 | 0% |  | 0.0 | 0% |
|  | Clinical Officer | **8.0** | **20%** |  | **8.0** | **20%** |  | **8.0** | **20%** |  | **8.0** | **20%** |  | 0.0 | 0% |
|  | Nursing Officer | **15.0** | **30%** |  | **15.0** | **30%** |  | 0.0 | 0% |  | 0.0 | 0% |  | 0.0 | 0% |
|  | Nurse Midwife Technician | **15.0** | **30%** |  | **15.0** | **30%** |  | **15.0** | **60%** |  | **15.0** | **60%** |  | **15.0** | **60%** |
|  | Medical Assistant | 0.0 | 0% |  | 0.0 | 0% |  | **15.0** | **20%** |  | **5.0** | **20%** |  | **15.0** | **20%** |
|  | Pharmacist | 0.0 | 0% |  | 0.0 | 0% |  | 0.0 | 0% |  | 0.0 | 0% |  | 0.0 | 0% |
|  | Pharmacy Technician | 0.0 | 0% |  | 0.0 | 0% |  | 0.0 | 0% |  | 0.0 | 0% |  | 0.0 | 0% |
|  | Pharmacy Assistant | 0.0 | 0% |  | 0.0 | 0% |  | 0.0 | 0% |  | 0.0 | 0% |  | 0.0 | 0% |
|  |  |  |  |  |  |  |  |  |  |  |  |  |  |  |  |
| *Accidents and Emergencies* | Medical Officer | **30.0** | **40%** |  | **30.0** | **20%** |  | 0.0 | 0% |  | 0.0 | 0% |  | 0.0 | 0% |
|  | Clinical Officer | **30.0** | **80%** |  | **30.0** | **80%** |  | **15.0** | **40%** |  | **15.0** | **40%** |  | 0.0 | 0% |
|  | Nursing Officer | **25.0** | **50%** |  | **25.0** | **50%** |  | 0.0 | 0% |  | 0.0 | 0% |  | 0.0 | 0% |
|  | Nurse Midwife Technician | **25.0** | **50%** |  | **25.0** | **50%** |  | **25.0** | **100%** |  | **25.0** | **100%** |  | **25.0** | **100%** |
|  | Medical Assistant | **20.0** | **100%** |  | **20.0** | **100%** |  | **15.0** | **60%** |  | **15.0** | **60%** |  | **15.0** | **100%** |
|  | Pharmacist | 0.0 | 0% |  | 0.0 | 0% |  | 0.0 | 0% |  | 0.0 | 0% |  | 0.0 | 0% |
|  | Pharmacy Technician | 0.0 | 0% |  | 0.0 | 0% |  | 0.0 | 0% |  | 0.0 | 0% |  | 0.0 | 0% |
|  | Pharmacy Assistant | 0.0 | 0% |  | 0.0 | 0% |  | 0.0 | 0% |  | 0.0 | 0% |  | 0.0 | 0% |
|  |  |  |  |  |  |  |  |  |  |  |  |  |  |  |  |
| *Major Surgical Procedures* | Medical Officer | **172.0** | **100%** |  | **172.0** | **100%** |  | 0.0 | 0% |  | 0.0 | 0% |  | 0.0 | 0% |
|  | Clinical Officer | **190.0** | **100%** |  | **190.0** | **100%** |  | **400.0** | **100%** |  | 0.0 | 0% |  | 0.0 | 0% |
|  | Nursing Officer | **172.0** | **80%** |  | **172.0** | **50%** |  | 0.0 | 0% |  | 0.0 | 0% |  | 0.0 | 0% |
|  | Nurse Midwife Technician | **172.0** | **20%** |  | **172.0** | **50%** |  | **172.0** | **100%** |  | 0.0 | 0% |  | 0.0 | 0% |
|  | Medical Assistant | 0.0 | 0% |  | 0.0 | 0% |  | 0.0 | 0% |  | 0.0 | 0% |  | 0.0 | 0% |
|  | Pharmacist | **10.0** | **50%** |  | 0.0 | 0% |  | 0.0 | 0% |  | 0.0 | 0% |  | 0.0 | 0% |
|  | Pharmacy Technician | **10.0** | **50%** |  | **10.0** | **100%** |  | **10.0** | **100%** |  | 0.0 | 0% |  | 0.0 | 0% |
|  | Pharmacy Assistant | 0.0 | 0% |  | 0.0 | 0% |  | 0.0 | 0% |  | 0.0 | 0% |  | 0.0 | 0% |
|  |  |  |  |  |  |  |  |  |  |  |  |  |  |  |  |
| *Minor Surgical Procedures* | Medical Officer | **60.0** | **100%** |  | **60.0** | **100%** |  | 0.0 | 0% |  | 0.0 | 0% |  | 0.0 | 0% |
|  | Clinical Officer | **80.0** | **100%** |  | **80.0** | **100%** |  | **200.0** | **100%** |  | 0.0 | 0% |  | 0.0 | 0% |
|  | Nursing Officer | **60.0** | **30%** |  | **60.0** | **30%** |  | **100.0** | **100%** |  | 0.0 | 0% |  | 0.0 | 0% |
|  | Nurse Midwife Technician | **60.0** | **70%** |  | **60.0** | **70%** |  | 0.0 | 0% |  | 0.0 | 0% |  | 0.0 | 0% |
|  | Medical Assistant | 0.0 | 0% |  | 0.0 | 0% |  | 0.0 | 0% |  | 0.0 | 0% |  | 0.0 | 0% |
|  | Pharmacist | **5.0** | **50%** |  | 0.0 | 0% |  | 0.0 | 0% |  | 0.0 | 0% |  | 0.0 | 0% |
|  | Pharmacy Technician | **5.0** | **50%** |  | **5.0** | **100%** |  | **5.0** | **100%** |  | 0.0 | 0% |  | 0.0 | 0% |
|  | Pharmacy Assistant | 0.0 | 0% |  | 0.0 | 0% |  | 0.0 | 0% |  | 0.0 | 0% |  | 0.0 | 0% |
|  |  |  |  |  |  |  |  |  |  |  |  |  |  |  |  |
| *TB Program - New Patient* | Medical Officer | **15.0** | **30%** |  | 0.0 | 0% |  | 0.0 | 0% |  | 0.0 | 0% |  | 0.0 | 0% |
|  | Clinical Officer | **15.0** | **30%** |  | **15.0** | **50%** |  | **15.0** | **50%** |  | **15.0** | **50%** |  | 0.0 | 0% |
|  | Nursing Officer | **20.0** | **40%** |  | **20.0** | **50%** |  | **20.0** | **50%** |  | **20.0** | **50%** |  | **20.0** | **25%** |
|  | Nurse Midwife Technician | **20.0** | **25%** |  | **20.0** | **25%** |  | **20.0** | **25%** |  | **20.0** | **25%** |  | **20.0** | **25%** |
|  | Medical Assistant | 0.0 | 0% |  | **15.0** | **25%** |  | **15.0** | **25%** |  | **15.0** | **25%** |  | **15.0** | **50%** |
|  | Pharmacist | 0.0 | 0% |  | 0.0 | 0% |  | 0.0 | 0% |  | 0.0 | 0% |  | 0.0 | 0% |
|  | Pharmacy Technician | **7.0** | **50%** |  | **7.0** | **50%** |  | 0.0 | 0% |  | 0.0 | 0% |  | 0.0 | 0% |
|  | Pharmacy Assistant | **7.0** | **50%** |  | **7.0** | **50%** |  | **7.0** | **100%** |  | **7.0** | **100%** |  | **7.0** | **100%** |
|  |  |  |  |  |  |  |  |  |  |  |  |  |  |  |  |
| *TB Program - Follow-up Patient* | Medical Officer | **10.0** | **25%** |  | 0.0 | 0% |  | 0.0 | 0% |  | 0.0 | 0% |  | 0.0 | 0% |
|  | Clinical Officer | **10.0** | **25%** |  | **10.0** | **25%** |  | **10.0** | **25%** |  | **10.0** | **25%** |  | 0.0 | 0% |
|  | Nursing Officer | **15.0** | **75%** |  | **15.0** | **80%** |  | **4.5** | **20%** |  | 0.0 | 0% |  | 0.0 | 0% |
|  | Nurse Midwife Technician | **10.5** | **50%** |  | 0.0 | 0% |  | 0.0 | 0% |  | 0.0 | 0% |  | 0.0 | 0% |
|  | Medical Assistant | 0.0 | 0% |  | **10.0** | **25%** |  | **10.0** | **25%** |  | **10.0** | **25%** |  | **10.0** | **50%** |
|  | Pharmacist | **3.0** | **50%** |  | **3.0** | **50%** |  | 0.0 | 0% |  | 0.0 | 0% |  | 0.0 | 0% |
|  | Pharmacy Technician | **3.0** | **50%** |  | **3.0** | **50%** |  | **3.0** | **100%** |  | **3.0** | **100%** |  | **3.0** | **100%** |
|  | Pharmacy Assistant | 0.0 | 0% |  | 0.0 | 0% |  | 0.0 | 0% |  | 0.0 | 0% |  | 0.0 | 0% |
|  |  |  |  |  |  |  |  |  |  |  |  |  |  |  |  |
| *Voluntary Counseling and Testing Program - For HIV-Negative* | Medical Officer | 0.0 | 0% |  | 0.0 | 0% |  | 0.0 | 0% |  | 0.0 | 0% |  | 0.0 | 0% |
|  | Clinical Officer | 0.0 | 0% |  | 0.0 | 0% |  | 0.0 | 0% |  | 0.0 | 0% |  | 0.0 | 0% |
|  | Nursing Officer | 0.0 | 0% |  | 0.0 | 0% |  | 0.0 | 0% |  | 0.0 | 0% |  | 0.0 | 0% |
|  | Nurse Midwife Technician | **20.0** | **100%** |  | **20.0** | **90%** |  | **20.0** | **90%** |  | **20.0** | **90%** |  | **20.0** | **80%** |
|  | Medical Assistant | 0.0 | 0% |  | 0.0 | 0% |  | 0.0 | 0% |  | 0.0 | 0% |  | 0.0 | 0% |
|  | Pharmacist | 0.0 | 0% |  | 0.0 | 0% |  | 0.0 | 0% |  | 0.0 | 0% |  | 0.0 | 0% |
|  | Pharmacy Technician | 0.0 | 0% |  | 0.0 | 0% |  | 0.0 | 0% |  | 0.0 | 0% |  | 0.0 | 0% |
|  | Pharmacy Assistant | 0.0 | 0% |  | 0.0 | 0% |  | 0.0 | 0% |  | 0.0 | 0% |  | 0.0 | 0% |
|  |  |  |  |  |  |  |  |  |  |  |  |  |  |  |  |
| *Voluntary Counseling and Testing Program - For HIV-Positive* | Medical Officer | 0.0 | 0% |  | 0.0 | 0% |  | 0.0 | 0% |  | 0.0 | 0% |  | 0.0 | 0% |
|  | Clinical Officer | 0.0 | 0% |  | 0.0 | 0% |  | 0.0 | 0% |  | 0.0 | 0% |  | 0.0 | 0% |
|  | Nursing Officer | 0.0 | 0% |  | 0.0 | 0% |  | 0.0 | 0% |  | 0.0 | 0% |  | 0.0 | 0% |
|  | Nurse Midwife Technician | **35.0** | **100%** |  | **35.0** | **90%** |  | **35.0** | **90%** |  | **35.0** | **90%** |  | **35.0** | **90%** |
|  | Medical Assistant | 0.0 | 0% |  | 0.0 | 0% |  | 0.0 | 0% |  | 0.0 | 0% |  | 0.0 | 0% |
|  | Pharmacist | 0.0 | 0% |  | 0.0 | 0% |  | 0.0 | 0% |  | 0.0 | 0% |  | 0.0 | 0% |
|  | Pharmacy Technician | 0.0 | 0% |  | 0.0 | 0% |  | 0.0 | 0% |  | 0.0 | 0% |  | 0.0 | 0% |
|  | Pharmacy Assistant | 0.0 | 0% |  | 0.0 | 0% |  | 0.0 | 0% |  | 0.0 | 0% |  | 0.0 | 0% |
|  |  |  |  |  |  |  |  |  |  |  |  |  |  |  |  |
| *Male Circumscisions* | Medical Officer | **20.0** | **50%** |  | 0.0 | 0% |  | 0.0 | 0% |  | 0.0 | 0% |  | 0.0 | 0% |
|  | Clinical Officer | **20.0** | **50%** |  | **20.0** | **100%** |  | **20.0** | **100%** |  | **20.0** | **100%** |  | 0.0 | 0% |
|  |  |  |  |  |  |  |  |  |  |  |  |  |  |  |  |
|  | Nurse Midwife Technician | **20.0** | **100%** |  | **20.0** | **100%** |  | **20.0** | **100%** |  | **20.0** | **100%** |  | 0.0 | 0% |
|  | Medical Assistant | 0.0 | 0% |  | 0.0 | 0% |  | 0.0 | 0% |  | 0.0 | 0% |  | 0.0 | 0% |
|  | Pharmacist | 0.0 | 0% |  | 0.0 | 0% |  | 0.0 | 0% |  | 0.0 | 0% |  | 0.0 | 0% |
|  | Pharmacy Technician | 0.0 | 0% |  | 0.0 | 0% |  | 0.0 | 0% |  | 0.0 | 0% |  | 0.0 | 0% |
|  | Pharmacy Assistant | 0.0 | 0% |  | 0.0 | 0% |  | 0.0 | 0% |  | 0.0 | 0% |  | 0.0 | 0% |
|  |  |  |  |  |  |  |  |  |  |  |  |  |  |  |  |
| *HIV/AIDS Program - New Adult* | Medical Officer | **15.0** | **40%** |  | 0.0 | 0% |  | 0.0 | 0% |  | 0.0 | 0% |  | 0.0 | 0% |
|  | Clinical Officer | **15.0** | **40%** |  | **15.0** | **40%** |  | **15.0** | **40%** |  | **10.0** | **40%** |  | 0.0 | 0% |
|  | Nursing Officer | **20.0** | **20%** |  | **20.0** | **25%** |  | 0.0 | 0% |  | 0.0 | 0% |  | 0.0 | 0% |
|  | Nurse Midwife Technician | **20.0** | **20%** |  | **20.0** | **25%** |  | **20.0** | **40%** |  | **20.0** | **40%** |  | **20.0** | **60%** |
|  | Medical Assistant | 0.0 | 0% |  | **15.0** | **25%** |  | **15.0** | **40%** |  | **15.0** | **40%** |  | **10.0** | **70%** |
|  | Pharmacist | **3.0** | **15%** |  | 0.0 | 0% |  | 0.0 | 0% |  | 0.0 | 0% |  | 0.0 | 0% |
|  | Pharmacy Technician | **2.0** | **50%** |  | **2.0** | **50%** |  | **2.0** | **50%** |  | **2.0** | **50%** |  | 0.0 | 0% |
|  | Pharmacy Assistant | **2.0** | **50%** |  | **2.0** | **50%** |  | **2.0** | **50%** |  | **2.0** | **50%** |  | **2.0** | **100%** |
|  |  |  |  |  |  |  |  |  |  |  |  |  |  |  |  |
| *HIV/AIDS Program - Established Medically Complex* | Medical Officer | **15.0** | **30%** |  | 0.0 | 0% |  | 0.0 | 0% |  | 0.0 | 0% |  | 0.0 | 0% |
|  | Clinical Officer | **15.0** | **50%** |  | **15.0** | **30%** |  | **15.0** | **30%** |  | **10.0** | **30%** |  | 0.0 | 0% |
|  | Nursing Officer | **15.0** | 0% |  | 0.0 | 0% |  | 0.0 | 0% |  | 0.0 | 0% |  | 0.0 | 0% |
|  | Nurse Midwife Technician | **15.0** | **40%** |  | **15.0** | **40%** |  | **14.0** | **40%** |  | **14.0** | **40%** |  | **14.0** | **45%** |
|  | Medical Assistant | 0.0 | 0% |  | **15.0** | **50%** |  | **15.0** | **50%** |  | **10.0** | **50%** |  | **10.0** | **75%** |
|  | Pharmacist | 0.0 | 0% |  | 0.0 | 0% |  | 0.0 | 0% |  | 0.0 | 0% |  | 0.0 | 0% |
|  | Pharmacy Technician | **1.5** | **50%** |  | **1.5** | **50%** |  | **1.5** | **50%** |  | **1.5** | **50%** |  | 0.0 | 0% |
|  | Pharmacy Assistant | **1.5** | **50%** |  | **1.5** | **50%** |  | **1.5** | **50%** |  | **1.5** | **50%** |  | **1.5** | **100%** |
|  |  |  |  |  |  |  |  |  |  |  |  |  |  |  |  |
| *HIV/AIDS Program - Established Non Medically Complex* | Medical Officer | 0.0 | 0% |  | 0.0 | 0% |  | 0.0 | 0% |  | 0.0 | 0% |  | 0.0 | 0% |
|  | Clinical Officer | **10.0** | **70%** |  | **10.0** | **25%** |  | **10.0** | **25%** |  | **10.0** | **5%** |  | 0.0 | 0% |
|  | Nursing Officer | **10.0** | 0% |  | 0.0 | 0% |  | 0.0 | 0% |  | 0.0 | 0% |  | 0.0 | 0% |
|  | Nurse Midwife Technician | **10.0** | **30%** |  | **10.0** | **50%** |  | **10.0** | **50%** |  | **10.0** | **90%** |  | **10.0** | **90%** |
|  | Medical Assistant |  |  |  | **10.0** | **25%** |  | **10.0** | **25%** |  | **10.0** | **5%** |  | **10.0** | **10%** |
|  | Pharmacist | 0.0 | 0% |  | 0.0 | 0% |  | 0.0 | 0% |  | 0.0 | 0% |  | 0.0 | 0% |
|  | Pharmacy Technician | **1.5** | **50%** |  | **1.5** | **50%** |  | **1.5** | **50%** |  | **1.5** | **50%** |  | 0.0 | 0% |
|  | Pharmacy Assistant | **1.5** | **50%** |  | **1.5** | **50%** |  | **1.5** | **50%** |  | **1.5** | **50%** |  | **1.5** | **100%** |
|  |  |  |  |  |  |  |  |  |  |  |  |  |  |  |  |
| *HIV/AIDS Program - PMTCT* | Medical Officer | **15.0** | **20%** |  |  |  |  | 0.0 | 0% |  | 0.0 | 0% |  | 0.0 | 0% |
|  | Clinical Officer | **15.0** | **20%** |  | **15.0** | **20%** |  | **15.0** | **20%** |  | **15.0** | **20%** |  | 0.0 | 0% |
|  | Nursing Officer | **20.0** | **50%** |  | 0.0 | 0% |  | 0.0 | 0% |  | 0.0 | 0% |  | 0.0 | 0% |
|  | Nurse Midwife Technician | **20.0** | **50%** |  | **20.0** | **100%** |  | **20.0** | **100%** |  | **20.0** | **50%** |  | **20.0** | **50%** |
|  | Medical Assistant | 0.0 | 0% |  | **15.0** | **20%** |  | **15.0** | **20%** |  | **15.0** | **20%** |  | **15.0** | **20%** |
|  | Pharmacist | **3.0** | **15%** |  | 0.0 | 0% |  | 0.0 | 0% |  | 0.0 | 0% |  | 0.0 | 0% |
|  | Pharmacy Technician | **2.0** | **50%** |  | **2.0** | **50%** |  | **2.0** | **50%** |  | **2.0** | **50%** |  | 0.0 | 0% |
|  | Pharmacy Assistant | **2.0** | **50%** |  | **2.0** | **50%** |  | **2.0** | **50%** |  | **2.0** | **50%** |  | **2.0** | **100%** |
|  |  |  |  |  |  |  |  |  |  |  |  |  |  |  |  |
| *HIV/AIDS Program – Pediatric* | Medical Officer | **15.0** | **20%** |  | 0.0 | 0% |  | 0.0 | 0% |  | 0.0 | 0% |  | 0.0 | 0% |
|  | Clinical Officer | **15.0** | **80%** |  | **15.0** | **80%** |  | **15.0** | **35%** |  | **15.0** | **30%** |  | 0.0 | 0% |
|  | Nursing Officer | **15.0** | **30%** |  | **15.0** | **40%** |  | 0.0 | 0% |  | 0.0 | 0% |  | 0.0 | 0% |
|  | Nurse Midwife Technician | **15.0** | 0% |  | 0.0 | 0% |  | **15.0** | **40%** |  | **15.0** | **40%** |  | **15.0** | **40%** |
|  | Medical Assistant | 0.0 | 0% |  | **15.0** | **20%** |  | **15.0** | **35%** |  | **15.0** | **30%** |  | **15.0** | **60%** |
|  | Pharmacist | 0.0 | 0% |  | 0.0 | 0% |  | 0.0 | 0% |  | 0.0 | 0% |  | 0.0 | 0% |
|  | Pharmacy Technician | **2.0** | **50%** |  | **2.0** | **50%** |  | **2.0** | **50%** |  | **2.0** | **50%** |  | 0.0 | 0% |
|  | Pharmacy Assistant | **2.0** | **50%** |  | **2.0** | **50%** |  | **2.0** | **50%** |  | **2.0** | **50%** |  | **2.0** | **100%** |

1. Percentage represents the division of labor between health workers of different cadres by showing what percentage of patients of each service type will be seen by a specific cadre. For example, for inpatient admission at a community hospital, all patients will be seen by a nurse midwife technician (30 minutes), a clinical officer (7 minutes), and a pharmacist (2 minutes), whereas at central and district hospitals more cadres may be involved in inpatient admissions but will not always see every patient (if the percentage for that cadre is less than 100%). A medical officer will see 25% of inpatient admissions at a district hospital.

| **Lab test type** | **Cadre** | **Central Hospital** | |  | **District Hospital** | |  | **Community Hospital** | |  | **Urban Health Center** | |  | | **Rural Health Center** | |
| --- | --- | --- | --- | --- | --- | --- | --- | --- | --- | --- | --- | --- | --- | --- | --- | --- |
|  |  | **Minutes** | **Percentage** |  | **Minutes** | **Percentage** |  | **Minutes** | **Percentage** |  | **Minutes** | **Percentage** |  | | **Minutes** | **Percentage** |
| *Haematology* | Lab Officer | **10.0** | **25%** |  | **10.0** | **25%** |  | 0.0 | 0% |  | 0.0 | 0% |  | | 0.0 | 0% |
|  | Lab Technician | **10.0** | **45%** |  | **10.0** | **45%** |  | **10.0** | **40%** |  | 0.0 | 0% |  | | 0.0 | 0% |
|  | Lab Assistant | **10.0** | **45%** |  | **10.0** | **45%** |  | **10.0** | **60%** |  | 0.0 | 0% |  | 0.0 | | 0% |
|  |  |  |  |  |  |  |  |  |  |  |  |  |  |  | |  |
| *POC* | Lab Officer | 0.0 | 0% |  | 0.0 | 0% |  | 0.0 | 0% |  | 0.0 | 0% |  | 0.0 | | 0% |
|  | Lab Technician | **10.0** | **50%** |  | **10.0** | **50%** |  | **10.0** | **50%** |  | **10.0** | **50%** |  | **10.0** | | **50%** |
|  | Lab Assistant | **10.0** | **50%** |  | **10.0** | **50%** |  | **10.0** | **50%** |  | **10.0** | **50%** |  | **10.0** | | **50%** |
|  |  |  |  |  |  |  |  |  |  |  |  |  |  |  | |  |
| *Parasitology* | Lab Officer | **15.0** | **25%** |  | **15.0** | **25%** |  | 0.0 | 0% |  | 0.0 | 0% |  | 0.0 | | 0% |
|  | Lab Technician | **15.0** | **45%** |  | **15.0** | **45%** |  | **15.0** | **40%** |  | 0.0 | 0% |  | 0.0 | | 0% |
|  | Lab Assistant | **15.0** | **45%** |  | **15.0** | **45%** |  | **15.0** | **60%** |  | 0.0 | 0% |  | 0.0 | | 0% |
|  |  |  |  |  |  |  |  |  |  |  |  |  |  |  | |  |
| *Biochemistry* | Lab Officer | **10.0** | **25%** |  | **10.0** | **25%** |  | 0.0 | 0% |  | 0.0 | 0% |  | 0.0 | | 0% |
|  | Lab Technician | **10.0** | **45%** |  | **10.0** | **45%** |  | **10.0** | **40%** |  | 0.0 | 0% |  | 0.0 | | 0% |
|  | Lab Assistant | **10.0** | **45%** |  | **10.0** | **45%** |  | **10.0** | **60%** |  | 0.0 | 0% |  | 0.0 | | 0% |
|  |  |  |  |  |  |  |  |  |  |  |  |  |  |  | |  |
| *Microbiology* | Lab Officer | **15.0** | **25%** |  | **15.0** | **25%** |  | 0.0 | 0% |  | 0.0 | 0% |  | 0.0 | | 0% |
|  | Lab Technician | **15.0** | **45%** |  | **15.0** | **45%** |  | 0.0 | 0% |  | 0.0 | 0% |  | 0.0 | | 0% |
|  | Lab Assistant | **15.0** | **45%** |  | **15.0** | **45%** |  | 0.0 | 0% |  | 0.0 | 0% |  | 0.0 | | 0% |
|  |  |  |  |  |  |  |  |  |  |  |  |  |  |  | |  |
| *Molecular* | Lab Officer | **10.0** | **100%** |  | 0.0 | 0% |  | 0.0 | 0% |  | 0.0 | 0% |  | 0.0 | | 0% |
|  | Lab Technician | **5.0** | **100%** |  | 0.0 | 0% |  | 0.0 | 0% |  | 0.0 | 0% |  | 0.0 | | 0% |
|  | Lab Assistant | **7.0** | **100%** |  | 0.0 | 0% |  | 0.0 | 0% |  | 0.0 | 0% |  | 0.0 | | 0% |
|  |  |  |  |  |  |  |  |  |  |  |  |  |  |  | |  |
| *TB Microscopy* | Lab Officer | **20.0** | **25%** |  | **20.0** | **25%** |  | 0.0 | 0% |  | 0.0 | 0% |  | 0.0 | | 0% |
|  | Lab Technician | **20.0** | **45%** |  | **20.0** | **35%** |  | **20.0** | **30%** |  | **20.0** | **30%** |  | **20.0** | | **30%** |
|  | Lab Assistant | **20.0** | **50%** |  | **20.0** | **60%** |  | **20.0** | **70%** |  | **20.0** | **70%** |  | **20.0** | | **70%** |
|  |  |  |  |  |  |  |  |  |  |  |  |  |  |  | |  |
| *Serology* | Lab Officer | **10.0** | **25%** |  | **10.0** | **25%** |  | 0.0 | 0% |  | 0.0 | 0% |  | 0.0 | | 0% |
|  | Lab Technician | **10.0** | **45%** |  | **10.0** | **50%** |  | **10.0** | **40%** |  | **10.0** | **40%** |  | **10.0** | | **40%** |
|  | Lab Assistant | **10.0** | **45%** |  | **10.0** | **50%** |  | **10.0** | **60%** |  | **10.0** | **60%** |  | **10.0** | | **60%** |
|  |  |  |  |  |  |  |  |  |  |  |  |  |  |  | |  |
| *Cytology* | Lab Officer | **10.0** | **100%** |  | 0.0 | 0% |  | 0.0 | 0% |  | 0.0 | 0% |  | 0.0 | | 0% |
|  | Lab Technician | **12.0** | **100%** |  | 0.0 | 0% |  | 0.0 | 0% |  | 0.0 | 0% |  | 0.0 | | 0% |
|  | Lab Assistant | **6.0** | **100%** |  | 0.0 | 0% |  | 0.0 | 0% |  | 0.0 | 0% |  | 0.0 | | 0% |
|  |  |  |  |  |  |  |  |  |  |  |  |  |  |  | |  |
| *Histology* | Lab Officer | **30.0** | **100%** |  | 0.0 | 0% |  | 0.0 | 0% |  | 0.0 | 0% |  | 0.0 | | 0% |
|  | Lab Technician | **10.0** | **100%** |  | 0.0 | 0% |  | 0.0 | 0% |  | 0.0 | 0% |  | 0.0 | | 0% |
|  | Lab Assistant | **5.0** | **100%** |  | 0.0 | 0% |  | 0.0 | 0% |  | 0.0 | 0% |  | 0.0 | | 0% |
|  |  |  |  |  |  |  |  |  |  |  |  |  |  |  | |  |
| *Blood Transfusion Lab Analysis* | Lab Officer | **15.0** | **25%** |  | **15.0** | **10%** |  | 0.0 | 0% |  | 0.0 | 0% |  | 0.0 | | 0% |
|  | Lab Technician | **15.0** | **45%** |  | **15.0** | **45%** |  | **15.0** | **40%** |  | 0.0 | 0% |  | 0.0 | | 0% |
|  | Lab Assistant | **15.0** | **45%** |  | **15.0** | **45%** |  | **15.0** | **60%** |  | 0.0 | 0% |  | 0.0 | | 0% |

Additional Table 5. Activity time assumptions for laboratory tests

Additional Table 6. Assumptions on patient facing time (available working time)

| **Item** | **Units** | **Medical Officer / Specialist** | **Clinical Officer / Technician** | **Medical Assistant** | **Nurse Officer** | **Nurse Midwife Technician** | **Pharmacist** | **Pharmacy Technician** | **Pharmacy Assistant** | **Lab Officer** | **Lab Technician** | **Lab Assistant** |
| --- | --- | --- | --- | --- | --- | --- | --- | --- | --- | --- | --- | --- |
| **DAYS PER YEAR** | | **M01** | **M02** | **M03** | **N01** | **N02** | **P01** | **P02** | **P03** | **L01** | **L02** | **L03** |
| Total Workdays per Year | days yearly | 260 | 260 | 260 | 260 | 260 | 260 | 260 | 260 | 260 | 260 | 260 |
| Public Holidays | days yearly | 11 | 11 | 11 | 11 | 11 | 11 | 11 | 11 | 11 | 11 | 11 |
| Annual Leave | days yearly | 26 | 24 | 24 | 24 | 24 | 24 | 24 | 24 | 24 | 24 | 24 |
| Bereavement Leave | days yearly | 3 | 2 | 3 | 8 | 7 | 3 | 3 | 3 | 2 | 2 | 2 |
| Sick Days (average taken) | days yearly | 14 | 2 | 3 | 14 | 7 | 1 | 1 | 1 | 1 | 1 | 1 |
| Maternity Leave |  |  |  |  |  |  |  |  |  |  |  |  |
| *Days taken per health worker on maternity leave* | days yearly | 90 | 90 | 90 | 90 | 90 | 90 | 90 | 90 | 90 | 90 | 90 |
| *Average days taken per year across cadre  (based on gender breakdown)* | days yearly | 1.6 | 1.9 | 2.2 | 5.4 | 5.6 | 1.3 | 1.3 | 1.3 | 1.0 | 1.0 | 1.0 |
| Training Days | days yearly | 8 | 3 | 3 | 2 | 2 | 3 | 3 | 3 | 3 | 3 | 3 |
| *Actual workdays per year after leave* | | 198 | 218 | 216 | 201 | 209 | 218 | 218 | 218 | 219 | 219 | 219 |
| . |  |  |  |  |  |  |  |  |  |  |  |  |
| **PATIENT FACING TIME PER DAY** | | **M01** | **M02** | **M03** | **N01** | **N02** | **P01** | **P02** | **P03** | **L01** | **L02** | **L03** |
| Facility-based working time on a normal day | hours daily | 7.0 | 7.0 | 7.0 | 7.0 | 7.0 | 7.0 | 7.0 | 7.0 | 7.0 | 7.0 | 7.0 |
| Average administrative or non-patient facing time per day | minutes daily | 140 | 60 | 60 | 200 | 120 | 90 | 90 | 90 | 90 | 80 | 60 |
| Average patient facing time per day | minutes daily | 280.00 | 360.00 | 360.00 | 220.00 | 300.00 | 330.00 | 330.00 | 330.00 | 330.00 | 340.00 | 360.00 |

**References**

1. Ministry of Health. National Community Health Strategy, 2017-2022. Lilongwe: Government of Malawi; 2017. <https://www.healthynewbornnetwork.org/resource/malawi-national-community-health-strategy-2017-2022/>.
2. Castro Lopes S, Guerra-Arias M, Pozo-Martin F, Nove A. A rapid review of the rate of attrition from the health workforce. Hum Resour Health. 2017; doi: 10.1186/s12960-017-0195-2.
3. Ministry of Health. Health sector strategic plan II: towards universal health coverage, 2017-2022. Lilongwe: Government of Malawi; 2017. <https://www.healthdatacollaborative.org/fileadmin/uploads/hdc/Documents/Country_documents/HSSP_II_Final_HQ_complete_file.pdf.pdf>.
4. Clinton Health Access Initiative. Malawi’s health workforce optimization analysis and report; a working paper: optimal allocation of health workers across Malawi’s public health facilities. 2014 (unpublished report).
5. World Bank Group. Draft final report of the analysis of human resources for health in Malawi through implementation of a WISN study in 75 facilities. Washington D.C.: World Bank Group; 2017. <https://openknowledge.worldbank.org/handle/10986/33307>.

1. The pipeline model assumes that this program launches in 2019. [↑](#footnote-ref-1)
